# Crosstalk between microRNA expression and DNA methylation drives the hormone-dependent phenotype of breast cancer

**DOI:** 10.1186/s13073-021-00880-4

**Published:** 2021-04-29

**Authors:** Miriam Ragle Aure, Thomas Fleischer, Sunniva Bjørklund, Jørgen Ankill, Jaime A. Castro-Mondragon, OSBREAC, Tone F. Bathen, Elin Borgen, Olav Engebråten, Olaf-Johan Hartman-Johnsen, Øystein Garred, Jürgen Geisler, Gry Aarum Geitvik, Solveig Hofvind, Anita Langerød, Ole Christian Lingjærde, Gunhild Mari Mælandsmo, Bjørn Naume, Hege G. Russnes, Helle Kristine Skjerven, Ellen Schlichting, Therese Sørlie, Anne-Lise Børresen-Dale, Jörg Tost, Kristine K. Sahlberg, Anthony Mathelier, Xavier Tekpli, Vessela N. Kristensen

**Affiliations:** 1 Department of Medical Genetics, Institute of Clinical Medicine, Faculty of Medicine, University of Oslo and Oslo University Hospital, Oslo, Norway; 2 Department of Cancer Genetics, Institute for Cancer Research, Oslo University Hospital, 0310 Oslo, Norway; 3 Centre for Molecular Medicine Norway (NCMM), Nordic EMBL Partnership, University of Oslo, 0318 Oslo, Norway; 4 Oslo Breast Cancer Research Consortium (OSBREAC), https://www.ous-research.no/home/kgjebsen/home/14105; 5 Institute for Clinical Medicine, University of Oslo, Oslo, Norway; 6 Laboratory for Epigenetics and Environment, Centre National de Recherche en Génomique Humaine, CEA–Institut de Biologie François Jacob, University Paris-Saclay, Evry, France; 7 Department of Research, Vestre Viken Hospital Trust, Drammen, Norway; 8 Department of Clinical Molecular Biology and Laboratory Science (EpiGen), Division of Medicine, Akershus University Hospital, Lørenskog, Norway

**Keywords:** miRNA, Methylation, Omics integration, Breast cancer, Systems biology

## Abstract

**Background:**

Abnormal DNA methylation is observed as an early event in breast carcinogenesis. However, how such alterations arise is still poorly understood. microRNAs (miRNAs) regulate gene expression at the post-transcriptional level and play key roles in various biological processes. Here, we integrate miRNA expression and DNA methylation at CpGs to study how miRNAs may affect the breast cancer methylome and how DNA methylation may regulate miRNA expression.

**Methods:**

miRNA expression and DNA methylation data from two breast cancer cohorts, Oslo2 (*n* = 297) and The Cancer Genome Atlas (*n* = 439), were integrated through a correlation approach that we term miRNA-methylation Quantitative Trait Loci (mimQTL) analysis. Hierarchical clustering was used to identify clusters of miRNAs and CpGs that were further characterized through analysis of mRNA/protein expression, clinicopathological features, in silico deconvolution, chromatin state and accessibility, transcription factor binding, and long-range interaction data.

**Results:**

Clustering of the significant mimQTLs identified distinct groups of miRNAs and CpGs that reflect important biological processes associated with breast cancer pathogenesis. Notably, two major miRNA clusters were related to immune or fibroblast infiltration, hence identifying miRNAs associated with cells of the tumor microenvironment, while another large cluster was related to estrogen receptor (ER) signaling. Studying the chromatin landscape surrounding CpGs associated with the estrogen signaling cluster, we found that miRNAs from this cluster are likely to be regulated through DNA methylation of enhancers bound by FOXA1, GATA2, and ER-alpha. Further, at the hub of the estrogen cluster, we identified hsa-miR-29c-5p as negatively correlated with the mRNA and protein expression of DNA methyltransferase DNMT3A, a key enzyme regulating DNA methylation. We found deregulation of hsa-miR-29c-5p already present in pre-invasive breast lesions and postulate that hsa-miR-29c-5p may trigger early event abnormal DNA methylation in ER-positive breast cancer.

**Conclusions:**

We describe how miRNA expression and DNA methylation interact and associate with distinct breast cancer phenotypes.

**Supplementary Information:**

The online version contains supplementary material available at 10.1186/s13073-021-00880-4.

## Background

Breast cancers are highly heterogeneous at the clinical and molecular level. Alterations of methylation at CpGs are found already in breast pre-invasive lesions [1] and are thought to shape the methylation patterns found in the different clinical and molecular breast cancer subtypes [2, 3]. The epigenome contributes to the cancer cells’ phenotype by regulating gene expression and the accessibility of regulatory regions. Previous studies have identified aberrant DNA methylation at gene promoters in breast cancer associated with clinically relevant subgroups. We recently showed that DNA methylation at enhancers identifies distinct breast cancer lineages [2]. The epigenome and the chromatin landscape are important features to explain breast cancer development and also progression, as recently demonstrated for endocrine resistance in breast cancer [4]. It is therefore essential to understand the crosstalk between the genome and the epigenome and its role in defining tumor phenotypes. Key enzymes, such as DNA methyltransferases (DNMTs) and ten-eleven translocation enzymes (TETs), regulate the DNA methylation machinery, and alterations of their expressions have been described in cancers with serious consequences in terms of cancer cell phenotype [5, 6]. However, how such enzymes may be early deregulated during carcinogenesis is still unclear.

MicroRNAs (miRNAs) are small (~ 22 nucleotides) non-coding RNAs regulating protein expression through targeting of messenger RNA (mRNA) for degradation or by inducing translational repression [7]. miRNAs play crucial roles in the regulation of cancer-associated processes such as proliferation, apoptosis, and differentiation and are known to elicit context and cell type-specific expression [8, 9]. In breast cancer, expression of miRNAs has been associated with clinical and molecular subtypes [10–12], progression [13–15], prognosis [16, 17], and expression of oncogenes [18]. Importantly, miRNAs have been shown to regulate the expression of epigenetic regulators such as DNMTs and TETs [19, 20]. Conversely, miRNA expression is regulated by DNA methylation of their respective promoters and aberrant methylation patterns of miRNA promoters has been associated with cancer [21–24]. We have previously shown how concerted alterations in copy number or promoter methylation affect miRNA expression *in cis*, resulting in upregulation of oncogenic miRNAs and downregulation of tumor-suppressor miRNAs [25]. Interestingly, methylation at regions flanking miRNA precursor sequences has recently been shown to impact miRNA expression and direct miRNA biogenesis [26]. However, how DNA methylation at distal regulatory regions is associated with miRNA expression in breast cancer remains poorly understood.

The aim of this study was to elucidate how miRNA expression associates with genome-wide DNA methylation patterns in breast cancer *in cis* (any association between a miRNA and CpG on the same chromosome) and *in trans* (any association between a miRNA and CpG on different chromosomes). Specifically, we studied the interplay between miRNA expression and DNA methylation and how it differs between breast cancer subtypes. To this end, we integrated whole-genome miRNA expression with CpG DNA methylation and performed a genome-wide correlation analysis identifying miRNA-methylation Quantitative Trait Loci (mimQTLs). We combined and integrated mimQTLs with mRNA/protein expression, clinicopathological information, ChromHMM genome segmentation, Assay for Transposase-Accessible Chromatin using sequencing (ATAC-seq) data, transcription factor (TF) binding, and long-range interaction data to elucidate miRNA-methylation crosstalk in breast cancer.

## Methods

### Clinical materials

Two independent breast cancer cohorts with DNA methylation and miRNA expression available were here used in parallel; the Oslo2 breast cancer cohort [17, 18] and The Cancer Genome Atlas Breast Invasive Carcinoma (TCGA-BRCA) cohort [12].

The Oslo2 breast cancer cohort has been previously described [17, 18] and is a consecutive study collecting material from breast cancer patients with primary operable disease at several hospitals in south-eastern Norway. Patients were included in the years 2006–2019. The study was approved by the Norwegian Regional Committee for Medical Research Ethics (approval number 1.2006.1607, amendment 1.2007.1125), and patients have given written consent for the use of material for research purposes.

The Illumina Infinium HumanMethylation450k microarray was used to measure the DNA methylation levels of more than 450,000 CpG sites for 330 patient tumors from the Oslo2 cohort as previously described [1, 27]. Each CpG probe returns a value called “beta” which is calculated as the methylated signal divided by the sum of the methylated and the unmethylated signal. The range of beta values is between 0 and 1 and thus represent the percentage of methylation of a given CpG site in the sample. Preprocessing and normalization involved steps of probe filtering, color bias correction, background subtraction, and subset quantile normalization. The DNA methylation data have been previously published [2]. For comparison of Oslo2 CpG DNA methylation levels to normal tissue, data from normal breast tissue from reduction mammoplasty (*n* = 17) were available [1].

The one-color microarray Human miRNA Microarray Kit (V2) design ID 029297 from Agilent Technologies was used to measure miRNA expression for 425 tumors of the Oslo2 cohort using 100 ng total RNA as input. Scanning was performed on the Agilent Scanner G2565A. Samples were processed using Feature Extraction version 10.7.3.1 (Agilent Technologies). The data were log_2_-transformed and for each tumor sample, considering only expressed miRNAs, the data were median centered. All non-expressed miRNAs across tumors were set to a common minimum value. The miRNA and mRNA expression data have been previously published [17]. In total, 297 Oslo2 tumor samples had matched methylation and miRNA expression data. Furthermore, of these, 45 samples had protein expression available measured by mass spectrometry and published in Johansson et al. [28].

The TCGA-BRCA cohort [12], from here on named TCGA, has been previously described [12]. For the DNA methylation data (level 3; beta values), probes with more than 50% missing values were removed, and further missing values were imputed using the function pamr.knnimpute (R package *pamr*) with *k* = 10. The log2(RPM + 1) miRNA mature strand expression data (level 3) measured by IlluminaHiseq were downloaded from the UCSC Xena browser [29]. In case of NAs, these were replaced with 0. Altogether, 439 TCGA breast cancer samples had matched methylation and miRNA expression data. In addition, DNA methylation level and miRNA expression data were available for 97 and 76 adjacent TCGA normal tissue samples, respectively. TCGA gene expression data in the form of log2(norm_count+ 1) measured by IlluminaHiseq_RNASeqV2 were downloaded from the UCSC Xena browser [29].

miRNA expression from ductal carcinoma in situ (DCIS) samples were available from Lesurf et al. [13]. In this data set, 26 DCIS samples and 14 invasive ductal carcinoma (IDC) had miRNA expression data, and out of these, 18 and 14, respectively, had estrogen receptor status available.

### Statistical and bioinformatical analysis

All analyses were performed in the R software v. 3.5.3 [30] unless otherwise specified. mimQTL analysis R code is available from GitHub: https://github.com/miriamragle/mimQTL.git [31].

### Genome-wide correlation analysis

Within each data set, CpGs with an interquartile range (IQR) > 0.1 and miRNAs expressed in > 10% of the samples were selected. Considering only CpGs and miRNAs present in both data sets resulted in 142,804 CpGs and 346 miRNAs (Additional file 1: Fig. S1). To test the correlation between the level of DNA methylation of CpGs and miRNA expression, the Spearman correlation statistics was applied (function *cor.test* with *method = “spearman”* in R). An association was considered statistically significant if a Bonferroni-corrected *p* value was < 0.05. Only significant correlations with the same direction (sign) were kept.

### Hierarchical clustering of mimQTLs

**Fig. 1:**
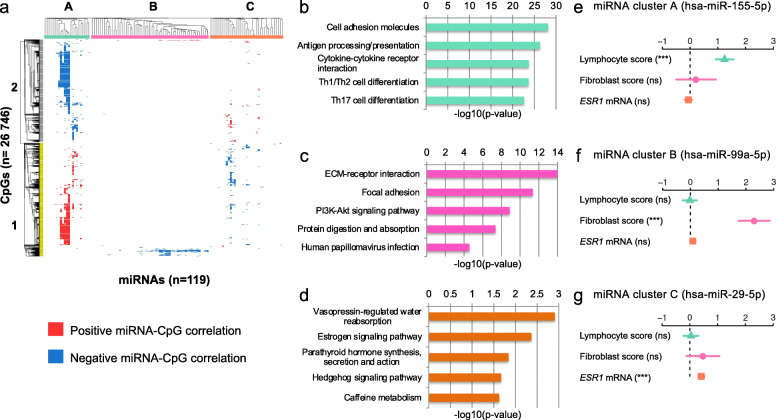
Identification of miRNA-methylation Quantitative Trait Loci (mimQTL) clusters and corresponding annotation. **a** Heatmap showing hierarchical clustering of the 89,118 significant mimQTLs found in both the Oslo2 and TCGA cohorts. miRNAs are shown in columns and CpGs in rows. In the heatmap, blue color indicates a negative correlation and red color indicates a positive correlation between miRNA expression and CpG methylation. Three main miRNA clusters (cluster A, B, and C) and two main CpG clusters (cluster 1 and 2) were identified. **b–d** Barplots showing the top five most enriched pathways for genes co-expressed (miRNA-mRNA expression Spearman correlation > 0.4) with the miRNAs of cluster A (**b**), B (**c**), and C (**d**). The *x*-axis show the − log10(*p* value) of the pathway enrichment obtained from Enrichr [33]. Bars are color-coded according to the associated miRNA cluster. **e–g** Results from fitting generalized linear models (GLM) to model miRNA expression as a multivariate function of lymphocyte infiltration (obtained by Nanodissect [34]), fibroblast infiltration (obtained by xCell [35]), and *ESR1* mRNA expression. The GLM coefficients are depicted with 95% confidence intervals for each of the miRNAs with the highest number of CpG associations in each cluster. Asterisks (***) denote a *p* value < 0.001 and “ns” denotes *not significant* (*p* value > 0.05)

The significant correlations overlapping the two data sets from the genome-wide correlation analysis were transformed into binary terms with − 1 for a significant negative correlation and + 1 for a significant positive correlation. The hierarchical clustering of CpGs and miRNAs was performed on these values using the R package *pheatmap* version 1.0.12 [32] with correlation distance and average linkage. CpGs and miRNAs with at least one significant association were included in the clustering analysis. To identify and decide upon the number of CpG and miRNA clusters, the dendrograms were visually inspected using different cut-offs for the *cutree_rows* and *cutree_cols* functions of the *pheatmap* package. Cut-offs were manually selected to define the clusters depicted in Fig. 1a (with cutree_rows = 2 and cutree_cols = 3). Manhattan plots of mimQTL miRNAs and CpGs were made using the R package *qqman* version 0.1.4 [36].

### Biological annotation of miRNA clusters

The expression of each miRNA in a given cluster was correlated to the mRNA expression of all genes. The miRNA-mRNA pairs that were positively (Spearman correlation > 0.4, *p* value < 0.05) and negatively correlated (Spearman correlation < − 0.3, *p* value < 0.05) in both the Oslo2 and TCGA cohorts were used in the downstream analysis (a negative threshold of < − 0.4 in both cohorts was too stringent and resulted in zero genes for miRNA cluster B). For each miRNA cluster, the list of positively or negatively correlated genes were selected and used as input to Enrichr [33] to perform gene set enrichment analysis (the analyses were performed on September 13th, 2019, and January 20th, 2021, for the positively and negatively correlated genes, respectively). Results obtained from the KEGG 2019 Human Pathways database were reported.

### Lymphocyte and fibroblast infiltration scores

The Nanodissect algorithm [34] (http://nano.princeton.edu/) was used for in silico estimation of lymphocyte infiltration as previously described [2]. The xCell algorithm [35] was used to obtain a fibroblast score for Oslo2 samples. For TCGA, xCell scores were downloaded from https://xcell.ucsf.edu/xCell_TCGA_RSEM.txt. To assess enrichment of positive or negative correlations between miRNA expression and infiltration scores for a given miRNA cluster, the *phyper* function in R was used with all miRNAs (*n* = 346) as background.

### miRNA expression modeled with generalized linear models

Generalized linear modeling (*glm* function in R) was used to model miRNA expression as a function of lymphocyte infiltration, fibroblast infiltration, and *ESR1* mRNA expression to estimate which variable(s) significantly associated with miRNA expression. The estimates plotted in Fig. 1e–g represent the multivariate analysis estimates with their 95% confidence intervals and the corresponding levels of significance (*p* values) are indicated.

### Pathway enrichment of genes mapped to mimQTL CpGs

For each of the CpGs in the two mimQTL CpG clusters, the corresponding gene was obtained by intersecting the Illumina450k array annotation file. The two gene lists were used as input to Enrichr [33] to perform gene set enrichment analysis (on 11.10.2019). As output, we exported the results from the KEGG 2019 Human Pathways database.

### Functional annotation of mimQTL CpGs

For functional annotation of the CpGs, we utilized the ChromHMM segmentation from Xi et al. [37] obtained from cell lines representing different breast cancer molecular subtypes [37]: MCF7 and ZR751 (luminal A), MB361 and UACC812 (luminal B), AU565 and HCC1954 (HER2) and MB469 and HCC1937 (basal). These segmentations were derived from Chromatin Immunoprecipitation Sequencing (ChIP-seq) data for five histone modification marks (H3K4me3, H3K4me1, H3K27me3, H3K9me3, and H3K36me3) to predict thirteen distinct chromatin states: active promoters (PrAct) and promoter flanking regions (PrFlk), active enhancers in intergenic regions (EhAct) and genic regions (EhGen), active transcription units (TxAct) and their flanking regions (TxFlk), strong (RepPC) and weak (WkREP) repressive polycomb domains, poised bivalent promoters (PrBiv) and bivalent enhancers (EhBiv), repeats/ZNF gene clusters (RpZNF), heterochromatin (Htchr), and quiescent/low signal regions (QsLow). We assessed enrichment of CpG sets within each of the 13 chromatin states using hypergeometric tests (the R function *phyper*) with all Illumina Infinium HumanMethylation450k BeadChip CpGs as background. *P* values were corrected using the Benjamini-Hochberg procedure [38].

Normalized ATAC-seq peak signals (log2((count+ 5)PM)-qn) for 74 TCGA breast tumors were downloaded from the Xena browser [29] (https://xenabrowser.net/datapages/). The CpG positions from the Illumina 450k array were intersected with the peaks using BEDTools v2.29.2 [39]. To test for differential open regions between estrogen receptor (ER)-positive and ER-negative tumors, the average normalized counts of the peaks containing each CpG within a CpG cluster was calculated per tumor and a Wilcoxon rank-sum test was applied to test for statistical significance using R.

### Enrichment of mimQTL CpGs at TF binding regions

**Fig. 2:**
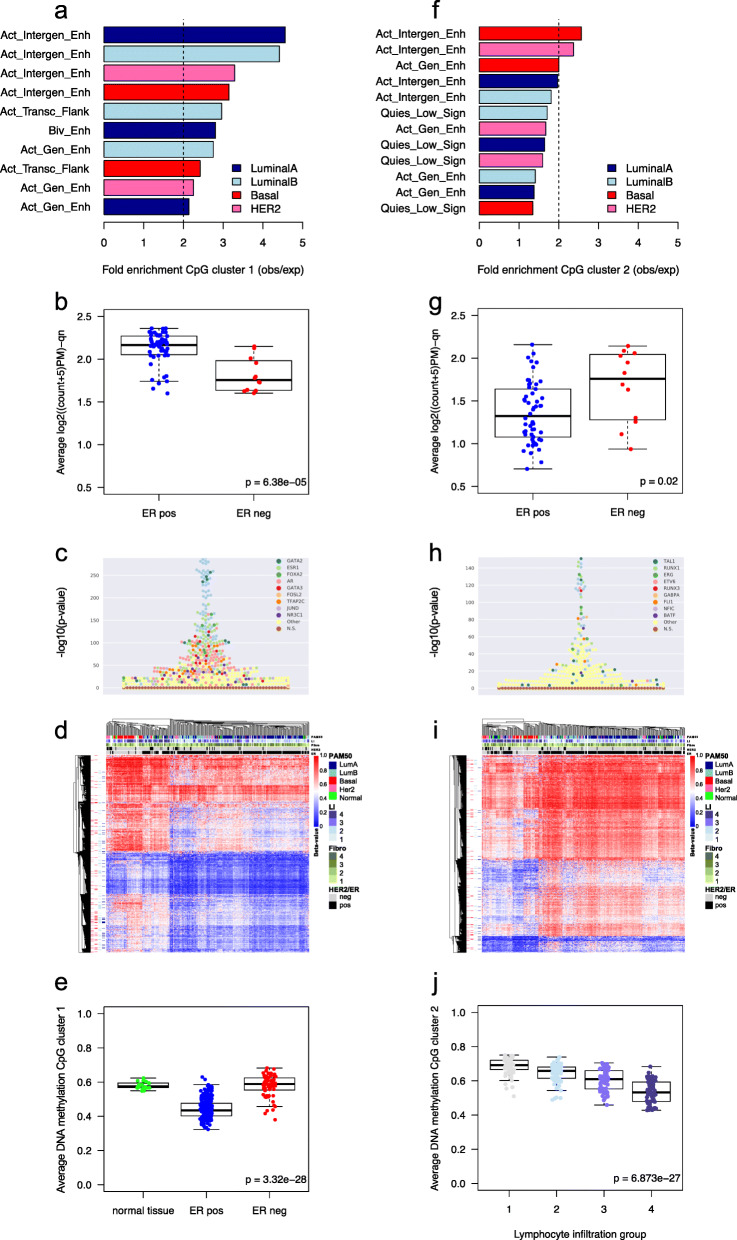
Functional annotation of the CpG clusters. **a** Genomic location enrichment of mimQTL CpGs in cluster 1 according to ChromHMM data from cell lines representing different breast cancer subtypes [37]. Only regions with fold-enrichment > 2 are shown. Active Genic Enhancer = Act_Gen_Enh, Active Transcription Flanking = Act_Transc_Flank, Bivalent Enhancer = Biv_Enh, Active Intergenic Enhancer = Act_Intergen_Enh, observed = obs, expected = exp. **b** Average normalized counts per tumor sample for all ATAC-seq peaks mapped to CpGs of cluster 1 (TCGA data). **c** Beeswarm plot showing enrichment of TF binding sites (−(log10(*p* value) using Fisher’s exact tests) on the *y*-axis for CpGs of cluster 1 (*n* = 14,040) according to UniBind [40]. TF names of the top 10 enriched TF binding sites data sets are provided with dedicated colors. Data sets for the same TFs are highlighted with the corresponding colors. **d** Heatmap showing hierarchical clustering of tumor methylation levels of CpG cluster 1 (*n* = 14,040) in the Oslo2 cohort (CpGs in rows and tumors in columns). Tumors are annotated according to PAM50 molecular subtypes; lymphocyte infiltration (LI) quartile groups 1(low)–4(high); fibroblast infiltration quartile groups (Fibro): 1(low)–4(high); human epidermal growth factor receptor 2 (HER2) status; estrogen receptor (ER) status. CpGs are annotated according to overlap with regions annotated as “Active Intergenic Enhancer” from ChromHMM of subtype-specific cell lines [37]; Her2 (pink), Basal (red), LumB (light blue), and LumA (dark blue). **e** Boxplot showing average DNA methylation of CpGs from cluster 1 in normal breast tissue (*n* = 17), ER-positive (pos; *n* = 223) and ER-negative tumors (neg; *n* = 60) of the Oslo2 cohort. **f** Enrichment of mimQTL CpGs in cluster 2 according to ChromHMM data. Quiescent_Low signals = Quies_Low_Sign. **g** Average normalized counts for ATAC-seq peaks mapped to CpGs of cluster 2. **h** Enrichment of TF binding sites for CpGs of cluster 2 (*n* = 12,706). **i** Hierarchical clustering of tumor methylation levels of CpG cluster 2 (*n* = 12,706). **j** Boxplot showing average DNA methylation of cluster 2 CpGs when Oslo2 tumors were separated into lymphocyte infiltration quartile groups from low (1) to high (4). Wilcoxon rank-sum *p* values (two-group comparisons) and Kruskal-Wallis *p* values (three or more groups) are indicated

To assess the enrichment of mimQTL CpGs close to transcription factor binding sites (TFBSs), we considered the direct TF-DNA interactions (i.e., TFBSs) stored in the UniBind database (version 1) [40] at https://unibind.uio.no. These TFBSs were obtained by combining both experimental (ChIP-seq) and computational (position weight matrices from JASPAR [41]) evidence of direct TF-DNA interactions (see Gheorghe et al. [40] for more details) for 231 TFs in 315 cell lines and tissues. Note that a TF can have multiple sets of TFBSs derived from different ChIP-seq experiments. The genomic positions of all CpGs from the Illumina 450k array were lifted over from hg19 to hg38 and extended with 100 bp on each side using BedTools (v2.26.0). The enrichment of UniBind TFBS sets in regions surrounding clusters 1 and 2 CpGs were assessed against a universe considering all CpG regions with the UniBind enrichment tool (https://unibind.uio.no/enrichment/ and https://bitbucket.org/CBGR/unibind_enrichment/). Specifically, the enrichment is computed using the LOLA R package (version 1.12.0) [42] using Fisher’s exact tests. Figure 2 c, h plots the Fisher’s exact *p* values using beeswarm plots (*swarmplot* function of the *seaborn* Python package, 10.5281/zenodo.824567) with annotations for the TFs associated with top 10 most enriched TFBS sets [43].

### Hierarchical clustering of methylation and miRNA expression

Hierarchical clustering of CpG DNA methylation or miRNA expression was performed using the R package *pheatmap* version 1.0.12 [32] with Euclidean distance and average linkage. For visualization, miRNA expression values were centered and scaled with *scale = “row”*.

### Statistical testing of methylation and miRNA expression between clinical/molecular groups

For two-group comparisons, Wilcoxon rank-sum tests were used considering a significance level of *p* < 0.05. For three or more groups, Kruskal-Wallis tests were used with the same significance level. When many tests were performed simultaneously, the resulting *p* values were corrected using the Benjamini-Hochberg procedure [38].

### Long-range interaction estimates and ChIP-seq peaks measurements

Chromatin Interaction Analysis by Paired-End Tag Sequencing (ChIA-PET) RNA polymerase II (Pol2) loop data from the MCF7 cell line was retrieved from ENCODE, accession number ENCSR000CAA [44]. Computational chromatin interactions predicted by the Integrated Methods for Predicting Enhancer Targets (IM-PET) algorithm [2] were retrieved from the 4Dgenome data portal for the ER-negative cell line HCC1954 [45]. The HiChIP-H3K27ac-DNA data for the ER-negative MDAMB231 cell line was obtained from GEO, accession number GSE97585 (samples GSM2572593 and GSM2572594) [46, 47]. The MDAMB231 data was converted from allvalidPairs.txt.gz files to bedpe format using an inbuilt script in the cLoops loop calling tool [48]. The output file was then directly processed using the cLoops loop calling algorithm with the default parameters (GitHub - YaqiangCao/cLoops: Accurate and flexible loops calling tool for 3D genomic data; https://github.com/YaqiangCao/cLoops. We investigated overlaps between long-range interaction loops and *in cis* (on the same chromosome) mimQTLs in R. A mimQTL (CpG-miRNA pair) were considered to be in a ChIA-PET loop if the CpG and the miRNA precursor were found in two different feet of the same loop. Enrichment was calculated using hypergeometric tests (*phyper* R function) with all possible *in cis* (i.e., on the same chromosome) pairs between miRNAs and CpGs of the 450k array as background. For the specific analyses of TF ChIP-seq data sets, we retrieved hg19 ENCODE ChIP-seq peak regions from the ReMap 2018 [49] database for the MCF7 and MDAMB231 cell lines (ENCSR000BST.GATA3.MCF7, ERP000783.ESR1.MCF7, GSE72249.FOXA1.MCF7, GSE66081.JUN.MDAMB231, and GSE48602.MYC.MDAMB231).

### miRNA super-enhancer (SE) breast tissue overlap with CpGs

miRNA SEs were retrieved from Suzuki et al. [50]. Data from the breast-associated cell lines HCC1954, HMEC, and MCF7 were considered. The overlap between CpG genomic positions and miRNA SEs was obtained using the *GenomicRanges* R package version 1.32.7 [51].

### Global methylation alteration (GMA) score

To obtain one score per tumor measuring the global methylation pattern deviation of a tumor from that of normal breast cells, a global methylation alteration (GMA) score was defined. Starting with the processed methylation data, for each CpG, the median beta value of all normal breast tissue samples (*n* = 17 for Oslo2 and *n* = 97 for TCGA, respectively) were calculated. The GMA score of a tumor *i* was then computed as:

$$ \mathrm{GMA}\ {\mathrm{score}}_i=\sum \left|\ {\mathrm{CpG}}_{j,i}-\mathrm{median}\left({\mathrm{CpG}}_{j, normals}\right)\ \right| $$

with CpG_*j,i*_ corresponding to the beta value of CpG *j* in tumor *i* and CpG_*j,*normals_ corresponding to the median of the beta values for CpG *j* in the normal breast tissue samples.

### In silico miRNA-target predictions

In silico-predicted miRNA-target interactions were downloaded from TargetScan release 7.2 [52] (http://www.targetscan.org/cgi-bin/targetscan/data_download.vert72.cgi), and both the Conserved and Nonconserved Site Context Scores were considered. From these predictions, we extracted the *Homo sapiens* in silico predictions for six selected genes: *TET1*, *TET2*, *TET3*, *DNMT1*, *DNMT3A*, and *DNMT3B*.

## Results

### Identification of miRNA-methylation quantitative trait loci (mimQTLs)

To identify robust associations between the expression of miRNAs and DNA methylation at CpG sites, we correlated genome-wide miRNA expression and DNA methylation in two independent breast cancer cohorts: Oslo2 (*n* = 297) and The Cancer Genome Atlas (TCGA) Breast Invasive Carcinoma cohort (*n* = 439; see Additional file 1: Fig. S1 for workflow outline). Only miRNAs and CpGs found in both cohorts were considered for estimation of the Spearman correlation between the expression of 346 miRNAs and methylation of 142,804 CpGs, resulting in 140,443 (0.28%) and 1,351,887 (2.74%) significant miRNA-CpG associations (Bonferroni-corrected *p* value < 0.05) in the Oslo2 and TCGA cohorts, respectively (see “Methods”). With a greater sample size for TCGA, a larger number of significant associations were observed, as expected (Additional file 1: Fig. S2). We identified 89,118 significant correlations with the same sign in both cohorts, pointing to consistent associations between miRNA expression and DNA methylation (Additional file 2), hereafter referred to as *miRNA-methylation Quantitative Trait Loci* (mimQTLs). These significant correlations involved 119 unique miRNAs and 26,746 unique CpGs (Additional file 3a, b). The observed correlations were more often negative than positive (64% negative vs. 36% positive: Additional file 1: Fig. S3a). A negative correlation in this context represents opposite trends, i.e., low CpG methylation and high miRNA expression (or vice versa), while a positive correlation represents the same trend, i.e., high CpG methylation is accompanied with high miRNA expression (or low CpG methylation and low miRNA expression). The genomic positions of the mimQTLs are displayed as Manhattan plots (Additional file 1: Fig. S3b). Although our analysis was not restricted to any distance parameter, a subset of mimQTLs (5125, 5.8%) were *in cis*, i.e., the miRNA and CpG were located on the same chromosome with the potential of direct functional interaction. The most significant *in cis* mimQTLs were found on chromosomes 1, 12, and 17 (Additional file 1: Fig. S3c). The frequency of negative *in cis* correlations was 63%, similar to the frequency across all mimQTLs. While causality cannot be directly inferred from such correlations, the mimQTL associations together reflect the global degree of coordination between CpG methylation and miRNA expression encompassing both direct and indirect interactions.

### Identification of mimQTL clusters

To identify mimQTLs sharing similar features with potential biological relevance, we performed unsupervised hierarchical clustering of the Spearman correlation *p* values binarized as − 1 (negative) and + 1 (positive), which led to the identification of three miRNA clusters (*x*-axis) and two CpG clusters (*y*-axis) (Fig. 1a). miRNA clusters A, B, and C consisted of 23, 59, and 37 miRNAs, respectively. CpG clusters 1 and 2 contained 14,040 and 12,706 CpGs, respectively. For each miRNA cluster, the number of associated mimQTL pairs were 66,202 (74% in cluster A), 9252 (11% in B), and 13,664 (15% in C). All clusters were characterized by predominantly negative correlations, but the fraction of negative to positive correlations varied between the miRNA clusters (Additional file 1: Fig. S3a). Of the CpGs associated with miRNAs in cluster C, 60% were also associated with miRNAs in cluster A but with the opposite sign of the correlation as indicated by the opposite blue or red colors on the heatmap (Fig. 1a). In contrast, most of the CpGs associated with miRNAs in cluster B were unique to this cluster. The number of CpG associations *per* miRNA varied from one up to 14,469 (hsa-miR-155-5p) with a median of 79 CpG associations (Additional file 1: Fig. S4a and Additional file 3 a). For the CpGs, the number of associations to miRNAs ranged from one up to 30 with a median of 3; only a small subset (1.2%) of almost exclusively cluster 1 CpGs had more than 10 miRNA associations (Additional file 1: Fig. S4b and Additional file 3b). As expected, a high degree of co-expression and co-methylation was observed by the members of a given cluster (Additional file 1: Fig. S5). These initial analyses led us to identify for the first time global and consistent correlations between miRNA expression and CpG methylation across two breast cancer cohorts.

### miRNA clusters highlight important processes of breast cancer pathogenesis

To identify biological functions shared by miRNAs in the same cluster (*x*-axis of the heatmap Fig. 1a), we identified genes positively co-expressed with the miRNAs of each cluster (mRNA-miRNA Spearman correlation > 0.4 in both cohorts; Additional file 3c) and performed gene set enrichment analyses (GSEA) using Enrichr [33]. We also performed the corresponding GSEA analysis on the negatively correlated genes separately (Additional file 3c).

#### miRNA cluster A—the immune cluster

miRNAs in cluster A were co-expressed with genes involved in immune cell differentiation and signaling and negatively correlated to genes associated with the estrogen signaling pathway (Fig. 1b and Additional file 3d). The top five miRNAs with most correlations to CpGs in cluster A (and also overall) were hsa-miR-155-5p (*n* = 14,469), hsa-miR-146a-5p (*n* = 12,546), hsa-miR-150-5p (*n* = 11,679), hsa-miR-142-5p (*n* = 8320), and hsa-miR-135b-5p (*n* = 5766). Concordant with the GSEA, we have previously shown in a third independent breast cancer cohort that these miRNAs are highly associated with immune response processes [10]. Importantly, previous studies have established functional roles for several of these miRNAs in immune cell differentiation and function [53–58]. To further confirm the association between miRNA cluster A and immune response, we used gene expression to score lymphocyte infiltration in each tumor using Nanodissect [34]. We found an enrichment of positive correlations between cluster A miRNA expression and tumor immune infiltration in both cohorts (hypergeometric test *p* value < 0.001 considering the correlation between all miRNAs and the lymphocyte score as background; Additional file 1: Fig. S6a, b and Additional file 3e). Altogether, these results suggest that miRNAs in cluster A are either expressed by tumor infiltrating immune cells or shape the tumor microenvironment. This is further supported by their higher expression in ER-negative tumors (Additional file 1: Fig. S7a, b and Additional file 3 f), which have higher immune infiltration compared to ER-positive tumors [59, 60].

#### miRNA cluster B—the fibroblast cluster

miRNAs in cluster B were co-expressed with genes enriched for extracellular matrix (ECM) and focal adhesion and negatively correlated to genes associated with cell cycle processes (Fig. 1c and Additional file 3d). As fibroblasts are strongly associated with biophysical forces of the tumor microenvironment and in shaping the ECM through the deposition of collagen [61], we computed a score reflecting the relative amount of fibroblasts in each sample using gene expression and the xCell [35] algorithm. We found that the expression of miRNAs in cluster B was significantly enriched for positive correlations to the fibroblast score (hypergeometric test *p* value < 0.001 considering the correlation between all miRNAs and the fibroblast score as background; Additional file 1: Fig. S6c, d and Additional file 3e). miRNAs of this cluster showed in general higher expression in ER-positive compared to ER-negative tumors of the Oslo2 cohort, and consistent differential expression between PAM50 subtypes with highest expression found in the luminal A and normal-like subtypes (Additional file 1: Fig. S7c, d and Additional file 3f). In support of these findings, a recent single-cell study observed enrichment of several fibroblast phenotypes among ER-positive/luminal breast tumors [62].

#### miRNA cluster C—the estrogen signaling cluster

miRNAs in cluster C were co-expressed with genes associated with hormone-regulated processes lead by estrogen signaling and negatively correlated to genes associated with cell cycle and immune-related pathways (Fig. 1d and Additional file 3d). Indeed, in both Oslo2 and TCGA the expression of cluster C miRNAs was significantly enriched for positive correlations to estrogen receptor mRNA (*ESR1*) expression (hypergeometric test *p* value < 0.001 considering the correlation between all miRNAs and *ESR1* mRNA as background; Additional file 1: Fig. S6e, f and Additional file 3e), and the miRNAs were mostly upregulated in ER-positive compared to ER-negative tumors (Additional file 1: Fig. S7e, f and Additional file 3f). We identified hsa-miR-29c-5p as the hub of cluster C with the highest number of associations to CpG methylation (*n* = 4764). This miRNA has been previously identified as one of the most significantly differentially expressed miRNAs between ER-positive and ER-negative tumors [10]. Thus, while miRNAs in cluster A and B reflect heterogeneity within the tumor microenvironment, miRNAs in cluster C are associated with estrogen signaling and ER-positive versus ER-negative breast cancer disease.

To further investigate the association between miRNA expression and the three characteristics of the clusters identified above (immune and fibroblast infiltration, and ER status), we modeled miRNA expression as a multivariate function of lymphocyte and fibroblast infiltration as well as *ESR1* mRNA expression (Additional file 3g). Figure 1e–g shows the coefficients for each characteristic to predict the expression of the miRNA with the highest number of CpG associations in each cluster. For nine out of 23 miRNAs in cluster A (Additional file 3 g), including hsa-miR-155-5p, the ‘hub’ of cluster A (Fig. 1e), the lymphocyte infiltration score was the most significant positive explanatory variable for expression (across both cohorts). In cluster B, the fibroblast infiltration score was significantly positively associated with miRNA expression for 51 out of 59 miRNAs across both cohorts as demonstrated for hsa-miR-99a-5p (Fig. 1f and Additional file 3g). For cluster C (Fig. 1g), *ESR1* mRNA expression (surrogate for ER status) was significantly positively associated with 15 out of 37 miRNAs, including hsa-miR-29c-5p expression, the hub of cluster C. Altogether, our analysis of miRNA expression in the three mimQTL-miRNA clusters clearly identified distinct signaling pathways and processes associated with different biological and molecular aspects of breast cancer.

### CpGs in mimQTL clusters reside in chromatin contexts associated with breast cancer subtypes

Next, we aimed to biologically annotate the CpGs of clusters 1 and 2, starting with their genomic position. First, we assessed pathway enrichment of their closest associated gene to infer any functional pathway association. Second, ChromHMM segmentation of the genome of several cell lines spanning breast cancer subtypes and ATAC-seq data was analyzed to study the genomic context of the CpGs within each cluster. Finally, we assessed their overlap with TFBSs derived from computational TF binding models and ChIP-seq data [40].

#### Cluster 1 CpGs

CpGs in cluster 1 mapped to 4809 genes according to the annotation of the Illumina HumanMethylation450k array. With GSEA using Enrichr [33], we found these genes enriched in signaling and cancer-associated pathways such as the Ras and PI3K-Akt signaling pathways (Additional file 3h). According to the ChromHMM genome segmentation of breast cancer cell lines [37], cluster 1 CpGs were enriched at enhancers, especially of ER-positive/luminal cell lines (Fig. 2a and Additional file 3i). ATAC-seq data from TCGA confirmed that the regions surrounding these CpGs were more accessible (open) in ER-positive than in ER-negative tumors (Wilcoxon *p* value = 6.38 × 10^− 5^; Fig. 2b). Furthermore, using the UniBind [40] database storing direct TF-DNA interactions for 231 TFs using 1983 human ChIP-seq data sets, we found cluster 1 CpGs enriched at FOXA1/2, GATA2/3, TFAP2C, and ESR1 (encoding ER-alpha) binding sites; these TFs are known to drive ER-positive breast cancers [2, 63] (Fig. 2c). Unsupervised clustering of the DNA methylation values associated with cluster 1 CpGs separated the tumors according to breast cancer subtypes (Fig. 2d and Additional file 1: Fig. S8a for the Oslo2 and TCGA cohorts, respectively). Cluster 1 CpGs showed overall lower DNA methylation in ER-positive and luminal breast cancer subtypes (Fig. 2e and Additional file 1: Fig. S9a). These lines of evidence show that cluster 1 CpGs are found at accessible enhancers with ER-associated TFBSs and are hypomethylated in ER-positive/luminal tumors.

#### Cluster 2 CpGs

Applying similar analyses to cluster 2 CpGs, we found the nearest genes (*n* = 3865) associated with cancer and immune system-related pathways (Additional file 3j). Cluster 2 CpGs were enriched at breast cancer enhancer regions, but to a lower extent than cluster 1 CpGs, and more at enhancers from ER-negative cell lines (Fig. 2f and Additional file 3i). Further, CpGs in cluster 2 were at genomic regions more accessible in ER-negative compared to ER-positive tumors, according to ATAC-seq data (Wilcoxon *p* value = 0.02; Fig. 2g). TFBSs associated with TFs involved in hematopoiesis and immune processes such as SPI1, TAL1, and RUNX1 were enriched close to cluster 2 CpGs (Fig. 2h). Unsupervised clustering using the DNA methylation of cluster 2 CpGs grouped breast cancer samples according to their level of lymphocyte infiltration derived from the Nanodissect scores (Fig. 2i). Finally, DNA methylation of cluster 2 CpGs negatively correlated with lymphocyte scores (i.e., low methylation–high lymphocyte infiltration and vice versa; Fig. 2j and Additional file 1: Fig. S9b). Thus, the methylation of cluster 2 CpGs is driven by intra-tumor heterogeneity characterized by infiltration of immune cells.

### Functional interpretation of CpG and miRNA mimQTL clusters

#### Functional association between CpG cluster 1 and miRNA cluster C

Altogether, we found cluster 1 CpGs to be associated with regulatory regions important for ER signaling and residing in more open and less-methylated genomic regions in ER-positive compared to ER-negative tumors (Fig. 2a, b). Hence, the negative correlations between CpG cluster 1 and miRNA cluster C observed in Fig. 1a represent functional CpG-miRNA associations of low methylation at cluster 1 CpGs (Fig. 2e) correlated with higher expression of cluster C miRNAs in ER-positive/luminal tumors (Additional file 3f).

#### Functional association between CpG cluster 2 and miRNA cluster A

Cluster 2 CpGs were associated with immune infiltration and negatively correlated through mimQTL with cluster A miRNAs (Fig. 1a), miRNAs themselves associated with immune infiltration [10]. We hypothesize that this observation is influenced by and reflect the variation in the presence of infiltrating immune cells in the tumors that have very different DNA methylation and miRNA expression than the cancer cells. To further support this interpretation, we retrieved DNA methylation data from ER-positive and ER-negative breast cancer cell lines and from different immune cell types (Additional file 1: Fig. S10). Focusing on the hub CpG of miRNA cluster A (the CpG with most associations to miRNAs in cluster A), a clear difference in methylation was observed between the cancer cell lines (hypermethylated) and immune cells (hypomethylated; Wilcoxon rank-sum *p* value = 4.56 × 10^− 12^). This is consistent with ER-negative/basal-like breast cancers showing higher immune infiltration compared to ER-positive/luminal tumors [60], as the methylation of cluster 2 CpGs was significantly lower (Additional file 1: Fig. S9c, d) and the miRNAs of cluster A were more expressed (Additional file 3f) in ER-negative/basal-like tumors.

### Regulatory networks encompassing DNA methylation and miRNA expression

#### Cluster 1 CpGs in miRNA super-enhancers

We next focused on the regulatory networks of miRNAs in cluster C as our mimQTL analysis highlighted regulatory regions (e.g., enhancers) linked to these miRNAs in a breast cancer subtype-specific manner. To find regulatory regions for miRNAs affected by DNA methylation, we targeted our analyses on the miRNA-CpG associations overlapping with (i) a catalog of super-enhancers (SE) recently identified by Suzuki et al. [50] to drive miRNA expression, (ii) experimentally derived long-range interactions with Pol2 binding (ChIA-PET Pol2 data) in the luminal MCF7 cell line [44], and (iii) binding regions for TFs known to drive ER-positive breast cancers (ER-alpha, FOXA1, and GATA3) in the MCF7 cell line (ChIP-seq data).

Altogether, 273 mimQTLs were identified where the CpG resides in an annotated breast miRNA SE (Additional file 3k). Interestingly, CpGs of cluster 1 were residing within miRNA SEs more often than the background of all CpGs (hypergeometric test *p* value = 1.29 × 10^− 26^), further confirming the enrichment of cluster 1 CpGs at distal regulatory regions regulating miRNA expression. Of the 273 mimQTLs, 50 represented direct *in cis* associations where the CpG was found in a miRNA SE mapping with the corresponding mimQTL miRNA [50]. These 50 *in cis* mimQTLs represented eight unique miRNAs all found to be cluster C miRNAs and all with significant negative correlations with the corresponding CpGs (Spearman correlation ranging from − 0.30 to − 0.68; Additional file 3k). This analysis underlines DNA methylation at super-enhancers as an important regulatory feature for miRNA expression in breast cancer.

**Fig. 3:**
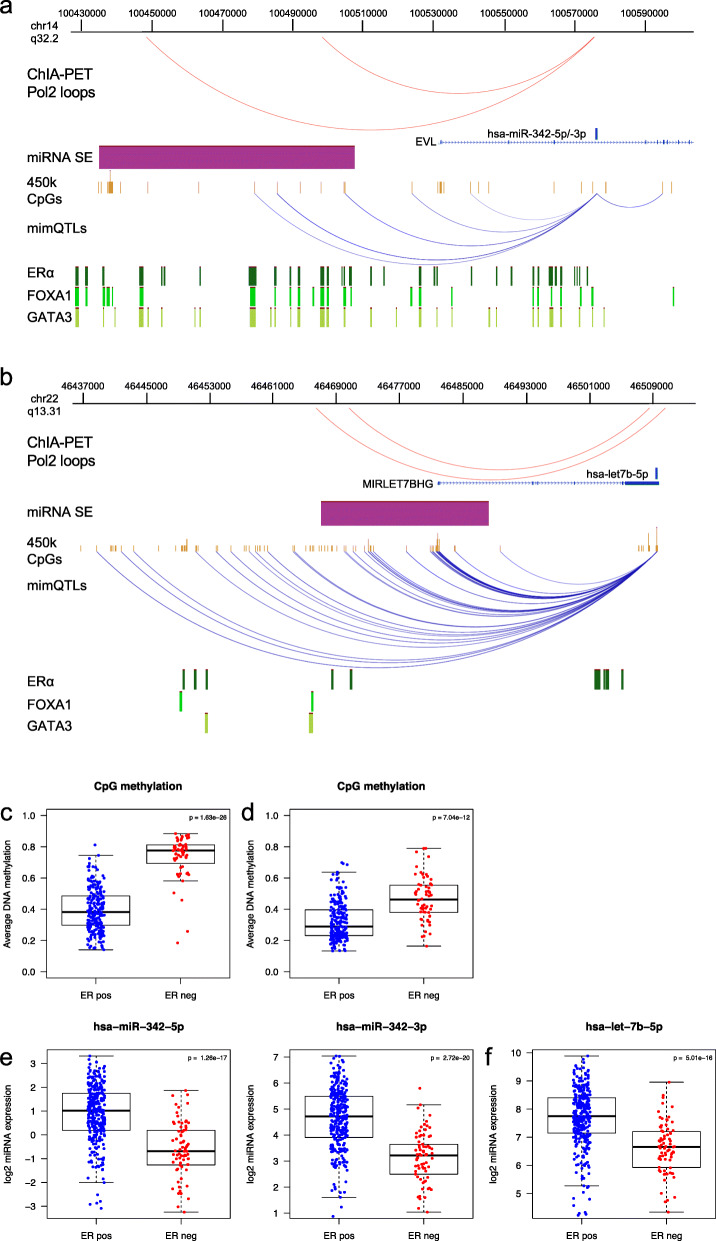
Super-enhancer (SE)–miRNA interactions and impact of CpG methylation on miRNA expression. **a** Example of mimQTLs (blue arcs) and ChIA-PET Pol2 loops (red arcs) where mimQTL CpGs (*n* = 3) or one foot of the ChIA-PET Pol2 loop is located within the hsa-miR-342 SE (purple) and the other loop foot resides within hsa-miR-342-5p/-3p. Also shown are the location of 450k methylation array CpGs and ER-alpha (ERα), FOXA1, and GATA3 binding regions obtained from ChIP-Seq experiments of the MCF7 cell line. The figure was made using the WashU Epigenome Browser v. 46.2 [64]. **b** mimQTLs and ChIA-PET Pol2 loops where mimQTL CpGs (*n* = 21) or one foot of the ChIA-PET Pol2 loop is located within the let-7b SE (purple) and the other loop foot resides within hsa-let-7b-5p. **c** Boxplot showing average DNA methylation in Oslo2 estrogen receptor (ER)-positive (pos) and ER-negative (neg) tumors across all CpGs within the hsa-miR-342 SE and in mimQTL with hsa-miR-342-3p/-5p (*n* = 3). **d** Boxplot showing average DNA methylation in Oslo2 ER-positive and ER-negative tumors across all CpGs within the let-7b SE and in mimQTL with hsa-let-7b-5p (*n* = 21). **e** Boxplots showing hsa-miR-342-5p/-3p expression in ER-positive and ER-negative tumors of the Oslo2 cohort. **f** Boxplots showing hsa-let-7b-5p expression in ER-positive and ER-negative tumors of the Oslo2 cohort. *P* values resulting from Wilcoxon rank-sum tests are indicated in the boxplots

*In cis* mimQTLs (i.e., CpG and miRNA on the same chromosome) were enriched at long-range chromatin interactions with Pol2 as defined by ChIA-PET in the luminal MCF7 cell line [35] (hypergeometric test *p* value = 4.51 × 10^− 4^). Two examples of overlap between ChiA-PET Pol2 loops, miRNA SE, and mimQTLs are shown for hsa-miR-342-3p/5p (Fig. 3a) and hsa-let7b-5p (Fig. 3b). We observed for these two examples that the SE/long-range interactions also overlap with TF binding regions for ER-alpha, FOXA1, or GATA3. The combination of these evidences suggests that through the mimQTL analysis we identify, for miRNA cluster C, hypomethylated regulatory regions in ER-positive breast cancer exemplified by hsa-miR-342-3p/5p (Fig. 3c) and hsa-let7b-5p (Fig. 3d), which may facilitate the binding of ER-alpha, FOXA1, and GATA3. This may further lead to the increased expression of hsa-miR-342-3p/5p (Fig. 3e) and hsa-let7b-5p (Fig. 3f) in ER-positive breast tumors. Altogether, these results suggest a direct regulatory link between the expression of cluster C miRNAs and DNA methylation at CpGs of ER-associated TF binding regions.

#### Long-range interaction loops in ER-negative breast cancer

We performed the same analysis in the context of ER-negative breast cancer using Integrated Methods for Predicting Enhancer Targets (IM-PET) data [65] from the ER-negative cell line HCC1954 and HiChIP-H3K27ac-DNA data (capturing chromatin interactions with active enhancer mark) from the ER-negative breast cancer cell line MDAMB231 [46, 47]. We reproduced the enrichment of *in cis* mimQTLs across long-range interaction loops in ER-negative breast cancer (hypergeometric test *p* values of 1.30 × 10^− 16^ and 1.23 × 10^− 12^ for HCC1954 and MDAMB231, respectively). The complete list of *in cis* mimQTL loops and the annotation with respect to selected transcription factor binding regions and long-range interactions is given in Additional file 3l. Potential enhancer–promoter loops common to the three long-range interaction data sets involved hsa-miR-196a-5p (miRNA cluster C) and hsa-miR-10a-5p (cluster B). Interestingly, hsa-let-7b-5p (cluster C) was overlapping with loops found both in the MCF7 and MDAMB231 cell lines. All three miRNAs showed higher expression in ER-positive compared to ER-negative tumors (Additional file 3f). In most cases, the mimQTLs found overlapping with long-range interactions represented negative correlation with lower methylation at CpGs associated with higher expression of miRNAs in ER-positive tumors compared to ER-negative tumors. Accordingly, while *in cis* long-range interactions were found present in both ER-positive and ER-negative cell lines, we hypothesize that the difference in transcription factor abundance and CpG methylation at the distal enhancer may be involved in tuning miRNA expression in tumors.

### miRNAs associated with global breast cancer DNA methylation alterations

**Fig. 4:**
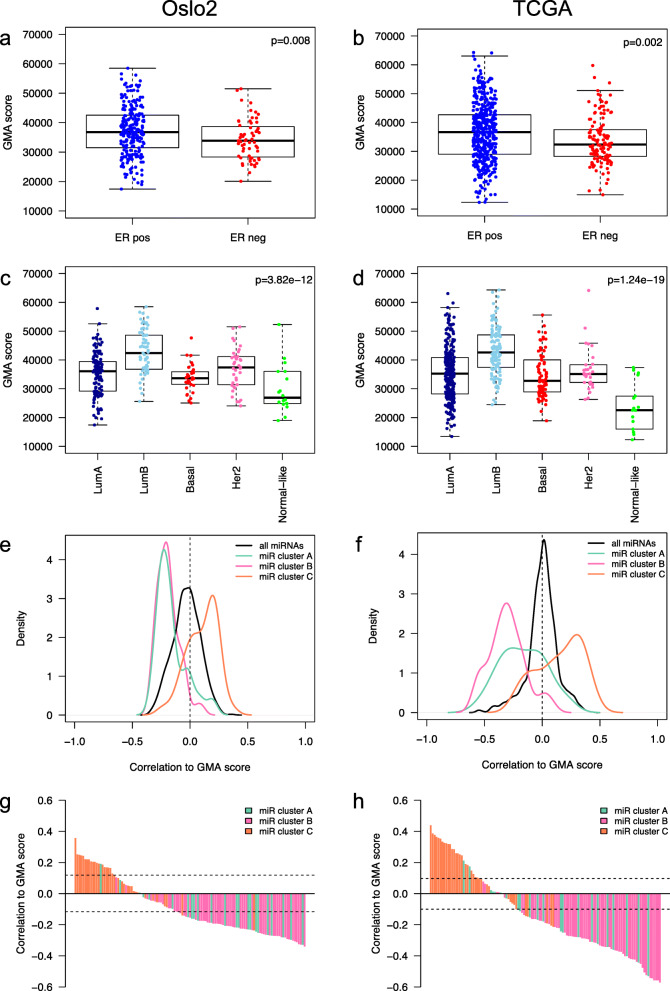
Global methylation alteration (GMA) score in clinical breast cancer groups and correlation to miRNA expression. **a, b** Boxplots showing the GMA score in estrogen receptor (ER)-positive (pos) and ER-negative (neg) tumors of the Oslo2 (**a**) and TCGA (**b**) cohorts. Wilcoxon rank-sum test *p* values are shown. **c, d** Boxplots showing the GMA score in PAM50 molecular subtypes of the Oslo2 (**c**) and TCGA (**d**) cohorts. LumA: Luminal A, LumB: Luminal B, Basal: Basal-like, Her2: HER2-enriched. Kruskal-Wallis test *p* values are denoted. **e, f** Plots showing density curves of the correlation between miRNA cluster members and the GMA score for the Oslo2 (**e**) and TCGA (**f**) cohorts. The density lines are color-coded according to miRNA cluster. **g, h** Barplots showing miRNAs decreasingly ranked according to GMA score correlation level (*y*-axis) in the Oslo2 (**g**) and TCGA (**h**) cohorts. The bars are color-coded according to miRNA cluster

Further, we sought to identify miRNAs associated with methylation deregulation in breast cancer. We developed a global methylation alteration (GMA) score that reflects how overall DNA methylation in breast tumors deviates from healthy breast tissue. In brief, for each tumor, the deviation in methylation per CpG relative to that of normal breast tissue was summed up (see “Methods” for details). We found that ER-positive including the luminal B tumors showed a higher GMA score than ER-negative tumors and other PAM50 subtypes (Fig. 4a–d). Additional file 1: Fig. S11 outlines the distribution of the GMA score in normal breast tissue samples compared to tumor samples showing how tumors have a higher and much broader GMA score compared to normal breast tissue. To identify which miRNAs may be the most potent at driving DNA methylation alterations, we correlated the expression of each of the 119 mimQTL miRNAs to the GMA score (Additional file 3 m). We observed that miRNAs in cluster C were enriched for positive correlations to the GMA score when compared to the background of all miRNAs tested (hypergeometric test *p* values < 0.001; Fig. 4e, f). Table 1 lists the miRNAs positively correlated with the GMA score across both cohorts. Inversely, cluster A and B miRNAs were enriched for negative correlations with the GMA score (hypergeometric test *p* values < 0.001). Such cluster-specific associations to the GMA score were further illustrated by plotting the correlation of each miRNA’s expression with the GMA score according to clusters (Fig. 4g, h).

**Table 1:** miRNAs significantly positively correlated with the global methylation alteration (GMA) score across both cohorts. The table is sorted according to correlations in the Oslo2 cohort

MIMAT	miRNA	miRNA cluster	Oslo2 Spearman correlation miRNA expression—GMA score	Oslo2 correlation ***p*** value	TCGA Spearman correlation miRNA expression—GMA score	TCGA correlation ***p*** value
MIMAT0003301	hsa-miR-33b-5p	C	0.357	2.30E−10	0.440	3.50E−22
MIMAT0002819	hsa-miR-193b-3p	C	0.252	1.18E−05	0.324	4.44E−12
MIMAT0000459	hsa-miR-193a-3p	C	0.249	1.47E−05	0.258	4.65E−08
MIMAT0000432	hsa-miR-141-3p	C	0.246	1.94E−05	0.358	1.35E−14
MIMAT0000728	hsa-miR-375	C	0.243	2.25E−05	0.286	1.20E−09
MIMAT0000095	hsa-miR-96-5p	C	0.221	1.19E−04	0.387	< 1.00E−25
MIMAT0000259	hsa-miR-182-5p	C	0.220	1.30E−04	0.326	3.40E−12
MIMAT0000682	hsa-miR-200a-3p	C	0.219	1.39E−04	0.246	2.01E−07
MIMAT0000688	hsa-miR-301a-3p	C	0.206	3.46E−04	0.318	1.11E−11
MIMAT0004673	hsa-miR-29c-5p	C	0.204	3.96E−04	0.333	1.00E−12
MIMAT0000261	hsa-miR-183-5p	C	0.204	4.04E−04	0.353	3.16E−14
MIMAT0000450	hsa-miR-149-5p	C	0.198	6.14E−04	0.317	1.36E−11
MIMAT0000617	hsa-miR-200c-3p	C	0.192	8.76E−04	0.365	3.58E−15
MIMAT0000252	hsa-miR-7-5p	A	0.192	8.95E−04	0.212	7.64E−06
MIMAT0003283	hsa-miR-615-3p	C	0.187	1.24E−03	0.252	9.96E−08
MIMAT0000267	hsa-miR-210	C	0.170	3.27E−03	0.377	1.65E−16
MIMAT0000710	hsa-miR-365a-3p	C	0.169	3.56E−03	0.260	3.76E−08
MIMAT0004929	hsa-miR-190b	C	0.164	4.49E−03	0.286	1.09E−09
MIMAT0004614	hsa-miR-193a-5p	C	0.128	2.75E−02	0.191	6.09E−05
MIMAT0005949	hsa-miR-664-3p	C	0.119	4.11E−02	0.148	1.86E−03

### hsa-miR-29c-5p is negatively correlated to DNA methyltransferase 3A and is deregulated early during breast cancer pathogenesis

**Fig. 5:**
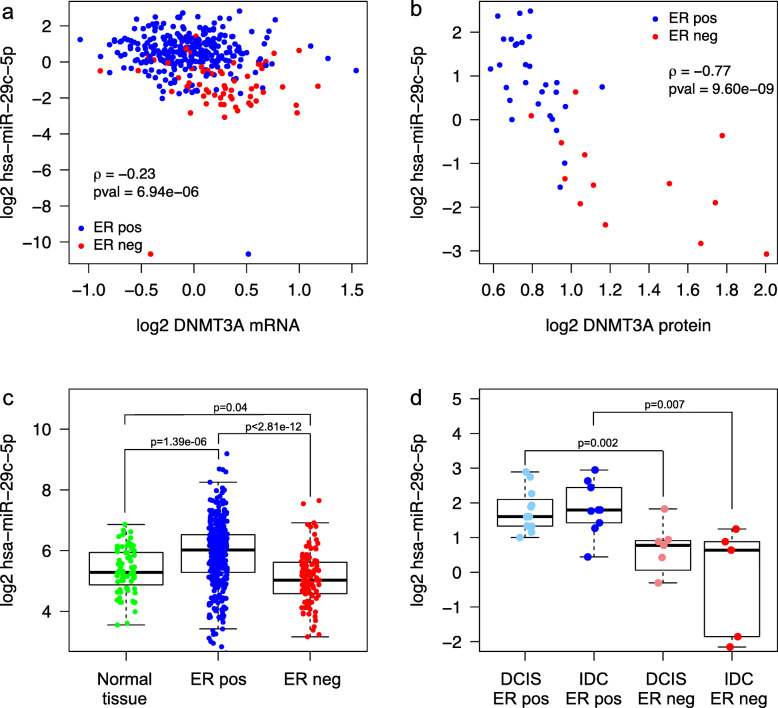
Expression of hsa-miR-29c-5p and correlation to DNMT3A. **a** DNMT3A mRNA expression (*x*-axis) vs. hsa-miR-29c-5p expression (*y*-axis) measured in 377 samples of the Oslo2 cohort. Estrogen receptor (ER)-positive (pos) tumors are plotted in blue and ER-negative (neg) in red. Spearman correlation coefficient (rho: ρ) and *p* value (pval) indicated. **b** DNMT3A protein expression (*x*-axis) vs. hsa-miR-29c-5p expression (*y*-axis) measured in 45 samples of the Oslo2 cohort. **c** hsa-miR-29c-5p expression in normal adjacent breast tissue (Normal tissue; *n* = 76), ER-positive (*n* = 333) and ER-negative tumors (*n* = 106) of the TCGA cohort. Wilcoxon rank-sum test *p* values are denoted. **d** hsa-miR-29c-5p expression in ER-positive (*n* = 11) and ER-negative (*n* = 7) ductal carcinoma in situ (DCIS) samples and ER-positive (*n* = 9) and ER-negative (*n* = 5) invasive ductal carcinoma (IDC) samples from the same data set [13]. Wilcoxon rank-sum test *p* values are denoted

Hypothesizing that cluster C miRNAs may be positively correlated with the GMA score through regulation of enzymes involved in DNA methylation, we queried the in silico target prediction database TargetScan [52] focusing on enzymes regulating DNA methylation (DNMTs and TETs). Altogether, 18 unique miRNAs belonging to miRNA cluster C were predicted to target DNMTs and/or TETs (Additional file 3n). We further found that three of the 18 miRNAs showed consistent and significant negative correlation to the mRNA of DNA methylation regulating enzymes (*DNMT3A* - hsa-miR-29c-5p (Fig. 5a), *TET1* - hsa-miR-365a-3p, and *TET1* - hsa-miR-375; Additional file 3n). We further confirmed the negative correlation between hsa-miR-29c-5p and DNMT3A also at the protein level (Fig. 5b, Spearman’s rho = − 0.77) using proteome data [28] of the Oslo2 samples (*n* = 45). This inverse correlation between hsa-miR-29c-5p and DNMT3A protein levels was the third most negative correlation considering all correlations between miRNAs (*n* = 713) and proteins (*n* = 9995) in the Oslo2 cohort. Of note, a significant negative correlation was also observed between hsa-miR-29c-5p and DNMT3B/DNMT1 mRNA and protein levels (Additional file 1: Fig. S12).

Furthermore, hsa-miR-29c-5p was found significantly upregulated in ER-positive tumors compared to normal breast tissue and ER-negative tumors (Wilcoxon rank-sum *p* value = 1.39 × 10^− 6^ and *p* value < 2.81 × 10^− 12^; Fig. 5c). To investigate whether the changes of hsa-miR-29c-5p expression may happen early during breast carcinogenesis causing DNA methylation alterations, we mined DCIS samples (*n* = 18) from an independent data set [13]. Indeed, in ER-positive pre-invasive DCIS lesions, hsa-miR-29c-5p was significantly more highly expressed than in ER-negative DCIS lesions (Wilcoxon rank-sum *p* value = 0.002; Fig. 5d), indicating that initial changes in hsa-miR-29c-5p expression may drive early DNA methylation changes in DCIS as previously observed [1].

## Discussion

This study is to our knowledge the first to assess the global relationship between the expression of miRNAs and DNA methylation on a genome-wide scale in breast cancer. Using two large and independent breast cancer cohorts, we have identified robust associations that point to how miRNA expression may be regulated through methylation at distal regulatory regions and how miRNAs may contribute to shape the epigenetic landscape of breast cancer. Further, the analysis points to how miRNA expression may reflect levels of infiltration from the surrounding microenvironment.

The methylation at a promoter CpG site and the expression of the corresponding gene is often observed to have a negative relationship, e.g., high methylation–low expression. With the increasing availability of methods to interrogate genome-wide methylation comprehensively at single-nucleotide resolution, the latter concept has been found to be more complex including positive correlations where methylation activates gene expression [66]. Overlapping the *in cis* mimQTL miRNAs with a catalog of miRNAs dysregulated in breast cancer due to aberrant methylation from a previous study [22], we found 40 of the *in cis* miRNAs (51%) associated with positive (35%) and negative (65%) CpG correlations. The overlap included miRNAs which expression has in additional studies been coupled to regulation by methylation such as hsa-miR-125b [23] and hsa-miR-195 [67]. This indicates that many of the *in cis* mimQTLs (CpG and miRNA on the same chromosome) may be direct functional associations. Interestingly, Glaich et al. [26] recently showed that when regions flanking the miRNA coding sequence are highly methylated, the miRNAs are higher expressed due to enhanced miRNA biogenesis. Coupling annotation from their study with the *in cis* mimQTLs (see Additional file 2), we observed for hsa-miR-338-3p and hsa-miR-452-5p positive correlation to CpGs flanking the miRNA genes, thus potentially identifying some examples of this phenomenon.

In this study, we go beyond CpG and miRNA genes linked by a threshold on distance and consider the correlation between *any* CpG and miRNA. With this comes the inherent challenge of separating direct from indirect associations. Utilizing additional layers of annotation and genomic data to further help the biological interpretation of the findings thus becomes necessary. The mimQTL approach identifies a positive or negative sign of the relation between a miRNA and a CpG, but does not infer the *causality* of the association, if any. The current approach does not take genetic variation into account such as single-nucleotide polymorphisms or copy number alterations, but this is something to be considered in future approaches.

Grouping of the mimQTLs using hierarchical clustering revealed aspects of underlying intra- and inter-tumor heterogeneity in the form of immune cell or fibroblast infiltration and tumor ER status. Expression of miRNAs in cluster A was positively associated with the lymphocyte score reflecting immune infiltration. Indeed, the miRNAs in this cluster with most CpG associations, hsa-miR-155-5p, hsa-miR-146a-5p, hsa-miR-150-5p, and hsa-miR-142-5p, have previously been associated with immune-related pathways [10] and lymphocytic infiltration [16] in breast cancer. Furthermore, in a study characterizing miRNA expression in various cell types and tissues from McCall et al. [9], these miRNAs were found to be most highly expressed in B and T cells, further suggesting that our miRNA cluster A reflect signals coming from infiltrating immune cells. This underlies a need for understanding more about which type of cells from bulk tumor samples are actually expressing nominated miRNA cancer biomarkers [68]. Importantly, the context- and cell type-specific expression of miRNAs give them an attractive potential for deconvolution tools. Using the deconvolution tool xCell [35] based on mRNA expression, we linked miRNAs in cluster B to fibroblast cells as their expression was positively correlated with the fibroblast score. Fibroblasts are providers of ECM components [69] and the genes co-expressed with the miRNAs were enriched for ECM-associated pathways. Assessing the catalog of cell- and tissue type-specific expression of miRNAs [9] supported this finding with the top miRNAs of this cluster, hsa-miR-99a-5p, hsa-miR-125b-5p, hsa-miR-379-5p, hsa-miR-381, and hsa-miR-100-5p, being highly expressed in tissue from skin where fibroblasts are a major component. Interestingly, hsa-miR-125b-5p was found to induce cardiac fibrosis [70]. Other studies pointed to a tumor-suppressor role of these miRNAs in breast cancer, for instance hsa-miR-99a-5p reduces breast cancer cell viability by targeting mTOR [71], hsa-miR-125b-5p was shown to induce cell cycle arrest and reduce cell growth in breast cancer cells [72], and hsa-miR-379-5p was shown to regulate Cyclin B1 expression [73]. More studies using for instance in situ hybridization of tumor tissue sections are needed to further validate the cells of origin of the immune- and fibroblast-associated miRNAs of cluster A and B, respectively, and will help to further refine the role of these miRNAs in breast cancer.

The miRNAs with most CpG associations in cluster C were markers of the ER-positive, luminal phenotype of breast cancer [10]. Integrating mimQTLs with data from various sources including long-range interaction loops, ATAC-seq, ChIP-seq, and miRNA SEs, we showed how miRNA expression may be promoted by ensuring open and active enhancer regions where ER-associated TFs bind and loop to the miRNA-encoding genomic regions boosting both their transcription and processing [50]. The corresponding connections in ER-negative tumors are more difficult to disentangle as the signal is to a larger degree a composite of tumor and immune cell infiltration [60]. With the identification of long-range interaction loops present in both ER-positive as well as ER-negative cell lines dominantly overlapping with miRNAs of cluster C, we postulate that differences in transcription factor abundance and DNA methylation at distal regulatory region CpGs may have a functional role affecting miRNA expression in tumors, but additional functional evidence is needed to conclude. We further hypothesize that the observed demethylation of the miRNA SE CpGs in ER-positive tumors lead to binding of ER-associated TFs making the SE active. Through activation, the SE is looped with Drosha/DGCR8—a protein complex important for processing of the primary miRNA transcript to the shorter precursor transcript [74]. This SE-mediated miRNA processing was previously shown with ChIP-seq peaks for DGCR8 observed at both the transcription start site (TSS) and precursor miRNA regions for SE-associated miRNAs [50]. The looping of the miRNA SE to the miRNA TSS or mature sequence boost the transcription and the processing of the miRNA [50], which was in our study further supported by the negative mimQTL correlation, i.e., low SE CpG methylation associated with high miRNA expression in ER-positive tumors. This emphasizes an important regulatory role for SE CpG methylation on miRNA expression in breast cancer. Indeed, miRNA expression deregulation in breast cancer through methylation alterations was previously described [16, 22, 25], but the focus has mainly been on CpGs in proximal promoter regions. As the importance of enhancer region conformation and methylation is becoming increasingly appreciated and given the great impact of miRNAs on the establishment and maintenance of cell phenotype, exploring this field will give new insights into cancer development and progression. Cluster C miRNAs were consistently enriched for positive correlations to the GMA score indicating that ER-positive, luminal tumors may be more severely altered at the methylation level compared to ER-negative tumors which may be more driven by alterations at the copy number level [12] and which showed methylation patterns more similar to the normal breast tissue samples.

Our analyses of the genomic positions and methylation of CpGs in each cluster highlighted CpG cluster 1 associated with differences in DNA methylation of enhancers and TFBS according to ER status. On the other hand, cluster 2 was associated with intra-tumor heterogeneity and infiltration of immune cells. Importantly, our CpG analyses mapped back to the biological functions associated with the miRNA clusters and therefore point to the fact that not only correlative but also functional associations link (i) CpG cluster 1 and miRNA cluster C as both being associated with estrogen response and ER status and (ii) CpG cluster 2 and miRNA cluster A being related to tumor immune infiltration. However, it is important to note that inter-tumor heterogeneity defined by ER status and intra-tumor heterogeneity defined by immune infiltration are at least partly two sides of the same coin as ER-negative tumors show a higher degree of immune infiltration [60].

We identified hsa-miR-29c-5p as a potential epigenetic hub in ER-positive breast cancer as it was the miRNA in cluster C with most CpG associations, positively correlated with the GMA score, upregulated in ER-positive tumors compared to both ER-negative tumors and normal breast tissue, in silico predicted to target DNMT3A and negatively correlated to DNMT3A mRNA and protein levels. Interestingly, the miR-29 family has previously been shown to directly target DNMTs [19, 75, 76], confirming a role for these miRNAs as epigenetic regulators. Fabbri et al. [19] showed in lung cancer a direct functional relationship of hsa-miR-29 family members directly targeting the 3′-UTR of DNMT3A/B. As we observe in breast tumors in our study, they also found significant anti-correlation between the levels of hsa-miR-29 family members and DNMT3A/B mRNA levels in lung tumors. Further functional validation in breast cancer cells is required to show the direct targeting of DNMT3A by hsa-miR-29c-5p and prove its causal role in determining luminal breast cancer phenotype. Supporting our hypothesis of hsa-miR-29c-5p being important for establishing the ER-positive/luminal breast cancer phenotype by targeting DNMT3A which leads to hypomethylation of CpGs at ER-associated TFBSs, Chou et al. [77] found that GATA3 acts as a TF inducing the expression of the miR-29 family. This and other studies have, however, pointed to a tumor-suppressor role of the miR-29 family in breast cancer as they are typically found more highly expressed in less aggressive/better prognosis subtypes and with over-expression in cell lines inhibiting metastasis, proliferation, migration, and growth [77–79]. Nevertheless, these findings are not contradictory with our hypothesis of the epigenetic regulator role of hsa-miR-29c-5p within luminal phenotypes. Importantly, focusing on subtype-specific progression, we previously found in an independent data set that hsa-miR-29c-5p is upregulated in expression from DCIS to luminal A and B tumors supporting the potential role of this miRNA in breast cancer progression within ER-positive tumors [14].

## Conclusions

In conclusion, we find that CpG methylation at ER-associated TF binding regions is likely to be important for regulation of miRNA expression in breast cancer. Furthermore, our study highlights that deregulation of hsa-miR-29c-5p expression is an early event that may result in downregulation of DNMT3A, which could further lead to hypomethylation of CpG sites important for ER-positive breast cancer cell identity. The CpG sites affected are at enhancer regions with TFBS for ER-alpha, FOXA1, and GATA3, all known to be important for the luminal breast cancer phenotype.

## Supplementary Information

**Additional file 1: Fig. S1.** Flowchart describing data and the different steps of the analysis leading to the identification of 89,118 miRNA-methylation Quantitative Trait Loci (mimQTLs). Examples of negative and positive correlation between methylation at a CpG and expression of a miRNA are shown as scatterplots at the bottom. **Fig. S2.** Overview of the 89,118 miRNA-CpG associations found significant in both cohorts. The scatterplots show a) the –log10(Bonferroni-adjusted Spearman correlation *p*-values) of Oslo2 (x-axis) vs. TCGA (y-axis); b) Spearman correlation coefficients in Oslo2 (x-axis) vs. TCGA (y-axis). The histograms show the distribution of the correlation coefficients (Spearman’s rho) of all significant miRNA-CpG correlations in the Oslo2 (c) and TCGA (d) cohorts. **Fig. S3.** Number of associations and genomic positions of mimQTL miRNAs and CpGs. a) Barplot showing the number of negative (neg) and positive (pos) CpG correlations (cor) for the three different miRNA clusters. b) mimQTL Manhattan plot with genomic coordinates of CpGs (black or gray) and miRNAs (green) displayed along the x-axis, with the negative logarithm of the Bonferroni-corrected Spearman correlation p-value from Oslo2 on the y-axis. Each dot on the plot signifies a CpG or miRNA (CpGs are shown in two colors to distinguish the chromosomes more clearly). c) *In cis* mimQTL Manhattan plot displaying the chromosomal location (using the position of the CpG) along the x-axis of the 5125 mimQTLs found on the same chromosome (*in cis*). Each dot represents one mimQTL which is color-coded according to negative (black) or positive (green) miRNA-CpG correlation. The y-axis displays the negative logarithm of the Bonferroni-corrected Spearman correlation *p*-value from Oslo2. **Fig. S4.** Barplots showing the number of associations per miRNA or CpG. a) Barplot showing the number of CpG associations per miRNA (*n* = 119). Note that the y-axis is on log scale. b) Barplot showing the number of miRNA associations per CpG (*n* = 26,746). **Fig. S5.** Density plots showing the degree of CpG co-methylation or miRNA co-expression between cluster members (see Fig. 1 and Additional file 3 a, b) calculated by Spearman correlation. a) Correlation between CpG cluster members in the Oslo2 data. b) Correlation between CpG cluster members in the TCGA data. c) Correlation between miRNA cluster members in the Oslo2 data. d) Correlation between miRNA cluster members in the TCGA data. The dotted lines represent density plots of corresponding correlations expected by chance, i.e. correlations observed after randomly permuting the same data before performing correlation analyses. **Fig. S6.** Density plots showing the distribution of Spearman correlation coefficients between miRNA expression and selected variables for members of each of the miRNA clusters. a, b) miRNA expression-immune infiltration score [34] correlations for the Oslo2 (a) and TCGA (b) cohorts. c, d) miRNA expression-fibroblast infiltration score [35] correlations for the Oslo2 (c) and TCGA (d) cohorts. e, f) miRNA expression-*ESR1* mRNA expression correlations for the Oslo2 (e) and TCGA (f) cohorts. **Fig. S7.** Heatmaps showing hierarchical clustering of miRNA expression levels (rows) from tumors (columns) of the Oslo2 (top) and TCGA (bottom) cohort. Clustering was performed using Euclidean distance and average linkage. Tumors are annotated with the following clinical/molecular classifications: PAM50 molecular subtypes (Luminal A (LumA), Luminal B (LumB), Basal-like (Basal), HER2-enriched (Her2), Normal-like (Normal); Lymphocyte infiltration (LI) group where tumors were divided into quartiles: 1 (low) – 4 (high); Fibroblast infiltration group (Fibro) where tumors were divided into quartiles: 1 (low) – 4 (high); Human epidermal growth factor receptor 2 (HER2) status; Estrogen receptor (ER) status. a, b) Clustering of miRNA cluster A expression (*n* = 23); c, d) Clustering of miRNA cluster B expression (*n* = 59); e, f) Clustering of miRNA cluster C expression (*n* = 37). **Fig. S8.** Heatmaps showing hierarchical clustering of methylation levels of CpG cluster 1 (a; *n* = 14,040) and CpG cluster 2 (b; *n* = 12,706) in the TCGA cohort (CpGs in rows and tumors in columns). Clustering was performed using Euclidean distance and average linkage. Tumors are annotated with the following clinical/molecular classifications: PAM50 molecular subtypes (Luminal A (LumA), Luminal B (LumB), Basal-like (Basal), HER2-enriched (Her2), Normal-like (Normal); Lymphocyte infiltration (LI) group where tumors were divided into quartiles: 1 (low) – 4 (high); Human epidermal growth factor receptor 2 (HER2) status; Estrogen receptor (ER) status. The CpGs are annotated according to overlap with regions annotated as “active intergenic enhancer” from ChromHMM of subtype-specific cell lines [37] with corresponding subtype colors. **Fig. S9.** Boxplot showing average DNA methylation of CpGs from cluster 1 in PAM50 subtypes of the Oslo2 cohort (Luminal A (LumA), Luminal B (LumB), Basal-like (Basal), HER2-enriched (Her2)). b) Boxplot showing average DNA methylation of CpGs from cluster 2 in the TCGA cohort when tumors were separated into quartile lymphocyte infiltration groups from low (1) to high (4) infiltration. c) Boxplot showing average DNA methylation of CpGs from cluster 2 in normal breast tissue (reduction mammoplasty, *n* = 17) or estrogen receptor (ER) positive (pos) or negative (neg) tumors of the Oslo2 cohort. d) Boxplot showing average DNA methylation of CpGs from cluster 2 in normal breast tissue (normal adjacent breast tissue, *n* = 97) or ER positive or negative tumors of the TCGA cohort. *P*-values resulting from Kruskal-Wallis tests indicated. **Fig. S10.** Boxplot showing DNA methylation of the hub CpG of miRNA cluster A (cg14270581; y-axis)) in ER positive (pos) and negative (neg) breast cancer cell lines and from different immune cell types (x-axis); B-cells, leukocytes (leuko), monocytes (mono) and T-cells. P-value resulting from Wilcoxon rank-sum test between cancer cell lines vs. immune cells is indicated. **Fig. S11.** Density plot showing the distribution of the Global Methylation Alteration (GMA) score in normal adjacent breast tissue (green), tumors (black) and tumors separated into estrogen receptor (ER) positive (pos) and negative (neg). Data from TCGA. **Fig. S12.** Top panel: Scatterplots showing on the x-axis mRNA expression of *DNMT3A* (left), *DNMT3B* (middle) and *DNMT1* (right) vs. hsa-miR-29c-5p expression (y-axis) measured in 377 samples of the Oslo2 cohort. Bottom panel: Scatterplots showing on the x-axis protein expression of DNMT3A (left), DNMT3B (middle) and DNMT1 (right) vs. hsa-miR-29c-5p expression (y-axis) measured in 45 samples of the Oslo2 cohort. Each dot represents a tumor color-coded according to PAM50 subtype (Luminal A (LumA): dark blue; Luminal B (LumB): light blue; Basal-like (Basal): red; HER2-enriched (Her2): pink; Normal-like: green). Spearman correlation coefficient and p-value are indicated for each plot.

**Additional file 2.** Table with annotation of the 89,118 mimQTLs found across the Oslo2 and TCGA cohorts. Genome locations are based on the hg19 build.

**Additional file 3.** a) Overview and annotation of the 119 mimQTL miRNAs. b) Overview and annotation of the 26,746 mimQTL CpGs. c) Genes found positively or negatively correlated to miRNAs of each cluster (mRNA-miRNA Spearman correlation > 0.4 or <-0.3, respectively, in both the Oslo2 and TCGA cohorts). d) Enrichr [33] Pathway enrichment (KEGG 2019 Human database) of genes positively (top) and negatively (bottom) correlated to miRNAs of each cluster. e) miRNA expression correlation to the Nanodissect [34] lymphocyte infiltration score, the xCell [35] fibroblast infiltration score, and *ESR1* mRNA expression. f) Differential expression of miRNAs between clinically relevant breast cancer groups. g) Results from generalized linear modeling (GLM) of miRNA expression as a multivariate function of lymphocyte and fibroblast infiltration and *ESR1* mRNA expression. h) Pathway enrichment using Enrichr [33] of genes mapped to the CpGs of cluster 1 according to the Illumina HumanMethylation450k array. i) ChromHMM [37] enrichment of genomic regions mapped to the CpGs of cluster 1 and 2. j) Pathway enrichment using Enrichr [33] of genes mapped to the CpGs of cluster 2 according to the Illumina HumanMethylation450k array. k) Table of 273 unique mimQTL associations with 69 unique CpGs residing in miRNA super-enhancer regions. l) Long-range loops overlapping with mimQTLs residing on the same chromosome. m) Correlation between the Global Methylation Alteration (GMA) score and 119 mimQTL miRNAs in the Oslo2 and TCGA cohorts. n) TargetScan [52] in silico predicted miRNA-target interactions for Global Methylation Alteration (GMA) score-correlated miRNAs and epigenetic regulator genes (conserved and non-conserved sites considered).

## Data Availability

mimQTL analysis R code is available from GitHub: https://github.com/miriamragle/mimQTL.git [31]. This study utilizes publicly available data sets: The DNA methylation data of the Oslo2 cohort are available from the Gene Expression Omnibus (GEO) database with accession number GSE84207, https://www.ncbi.nlm.nih.gov/geo/query/acc.cgi?acc=GSE84207 [2]. For comparison of Oslo2 CpG DNA methylation levels to normal tissue, data from normal breast tissue were available in GEO with accession number GSE60185, https://www.ncbi.nlm.nih.gov/geo/query/acc.cgi?acc=GSE60185 [1]. The Oslo2 miRNA expression is available from GEO with accession number GSE81000 (https://www.ncbi.nlm.nih.gov/geo/query/acc.cgi?acc=GSE81000) and mRNA expression from accession number GSE80999 (https://www.ncbi.nlm.nih.gov/geo/query/acc.cgi?acc=GSE80999) [17, 18]. These accessions also include Oslo2 clinical data. The Oslo2 protein data were deposited in the ProteomeXchange database under the accession code PXD008841, http://proteomecentral.proteomexchange.org/cgi/GetDataset?ID=PXD008841 [28]. For TCGA, the DNA methylation data (level 3) were downloaded from the TCGA Data Portal (https://tcga-data.nci.nih.gov). The TCGA miRNA and mRNA expression data (level 3), clinical data, and ATAC-seq data were downloaded from the UCSC Xena browser [29] (https://xenabrowser.net/datapages/). miRNA expression from DCIS samples together with clinical information were available from GEO data set GSE59248 (https://www.ncbi.nlm.nih.gov/geo/query/acc.cgi?acc=GSE59248) [13]. DNA methylation data from cancer cell lines and immune cells were collected from GEO with the following accession numbers: GSE94943 (breast cancer cell lines; https://www.ncbi.nlm.nih.gov/geo/query/acc.cgi?acc=GSE94943), GSE69270 (leukocytes; https://www.ncbi.nlm.nih.gov/geo/query/acc.cgi?acc=GSE69270) [80], GSE68456 (B-cells and monocytes; https://www.ncbi.nlm.nih.gov/geo/query/acc.cgi?acc=GSE68456) [81], and GSE79144 (T cells; https://www.ncbi.nlm.nih.gov/geo/query/acc.cgi?acc=GSE79144) [82]. ChromHMM segmentation of breast cancer cell lines was available through personal communication with Xi et al. [37]. Direct TF-DNA interactions were available from the UniBind database [40] at https://unibind.uio.no. ChIA-PET Pol2 loop data from the MCF7 cell line was retrieved from ENCODE, accession number ENCSR000CAA, https://www.encodeproject.org/experiments/ENCSR000CAA/ [44]. Computational chromatin interactions predicted by the IM-PET algorithm [2] was retrieved from the 4Dgenome data portal for the ER-negative cell line HCC1954 (https://4dgenome.research.chop.edu/Download.html) [45]. The HiChIP data was obtained from GEO, accession number GSE97585 (samples GSM2572593 and GSM2572594) (https://www.ncbi.nlm.nih.gov/geo/query/acc.cgi?acc=GSE97585) [46]. For the specific analyses of TF ChIP-seq data sets, we retrieved hg19 ENCODE ChIP-seq peak regions from the ReMap 2018 [49] database (http://remap.univ-amu.fr/download_page#remap2018tab) for the MCF7 and MDAMB231 cell lines (ENCSR000BST.GATA3.MCF7, ERP000783.ESR1.MCF7, GSE72249.FOXA1.MCF7, GSE66081.JUN.MDAMB231, and GSE48602.MYC.MDAMB231). miRNA SEs data were retrieved from Table S2 in Suzuki et al. [50].

## Abbreviations

ATAC-seq: Assay for Transposase-Accessible Chromatin using sequencing
Basal: Basal-like
ChIA-PET: Chromatin Interaction Analysis by Paired-End Tag Sequencing
ChIP-seq: Chromatin Immunoprecipitation Sequencing
DNMTs: DNA methyltransferases
DCIS: Ductal carcinoma in situ
ER: Estrogen receptor
ECM: Extracellular matrix
GEO: Gene Expression Omnibus
GLM: Generalized linear model
GMA: Global methylation alteration
Her2: HER2-enriched
IM-PET: Integrated Methods for Predicting Enhancer Targets
IQR: Interquartile range
IDC: Invasive ductal carcinoma
LumA: Luminal A
LumB: Luminal B
miRNA: MicroRNA
mRNA: Messenger RNA
mimQTL: miRNA-methylation Quantitative Trait Loci
neg: Negative
pos: Positive
Pol2: RNA polymerase II
SE: Super-enhancer
TCGA: The Cancer Genome Atlas
TCGA-BRCA: The Cancer Genome Atlas Breast Invasive Carcinoma
TETs: Ten-eleven translocation enzymes
TF: Transcription factor
TFBS: Transcription factor binding site
TSS: Transcription start site
