# Supplementary material for: Crosstalk between microRNA expression and DNA methylation drives the hormone-dependent phenotype of breast cancer
Source: Genome Med. 2021 Apr 29;13:72. doi: 10.1186/s13073-021-00880-4 (PMC8086068; doi:10.1186/s13073-021-00880-4)
Supplement: Supplementary file 1 — Additional file 1: Fig. S1. Flowchart describing data and the different steps of the analysis leading to the identification of 89,118 miRNA-methylation Quantitative Trait Loci (mimQTLs). Examples of negative and positive correlation between methylation at a CpG and expression of a miRNA are shown as scatterplots at the bottom. Fig. S2. Overview of the 89,118 miRNA-CpG associations found significant in both cohorts. The scatterplots show a) the –log10(Bonferroni-adjusted Spearman correlation p-values) of Oslo2 (x-axis) vs. TCGA (y-axis); b) Spearman correlation coefficients in Oslo2 (x-axis) vs. TCGA (y-axis). The histograms show the distribution of the correlation coefficients (Spearman’s rho) of all significant miRNA-CpG correlations in the Oslo2 (c) and TCGA (d) cohorts. Fig. S3. Number of associations and genomic positions of mimQTL miRNAs and CpGs. a) Barplot showing the number of negative (neg) and positive (pos) CpG correlations (cor) for the three different miRNA clusters. b) mimQTL Manhattan plot with genomic coordinates of CpGs (black or gray) and miRNAs (green) displayed along the x-axis, with the negative logarithm of the Bonferroni-corrected Spearman correlation p-value from Oslo2 on the y-axis. Each dot on the plot signifies a CpG or miRNA (CpGs are shown in two colors to distinguish the chromosomes more clearly). c) In cis mimQTL Manhattan plot displaying the chromosomal location (using the position of the CpG) along the x-axis of the 5125 mimQTLs found on the same chromosome (in cis). Each dot represents one mimQTL which is color-coded according to negative (black) or positive (green) miRNA-CpG correlation. The y-axis displays the negative logarithm of the Bonferroni-corrected Spearman correlation p-value from Oslo2. Fig. S4. Barplots showing the number of associations per miRNA or CpG. a) Barplot showing the number of CpG associations per miRNA (n = 119). Note that the y-axis is on log scale. b) Barplot showing the number of miRNA associations per Cp [file 13073_2021_880_MOESM1_ESM.pdf]

Fig. S1

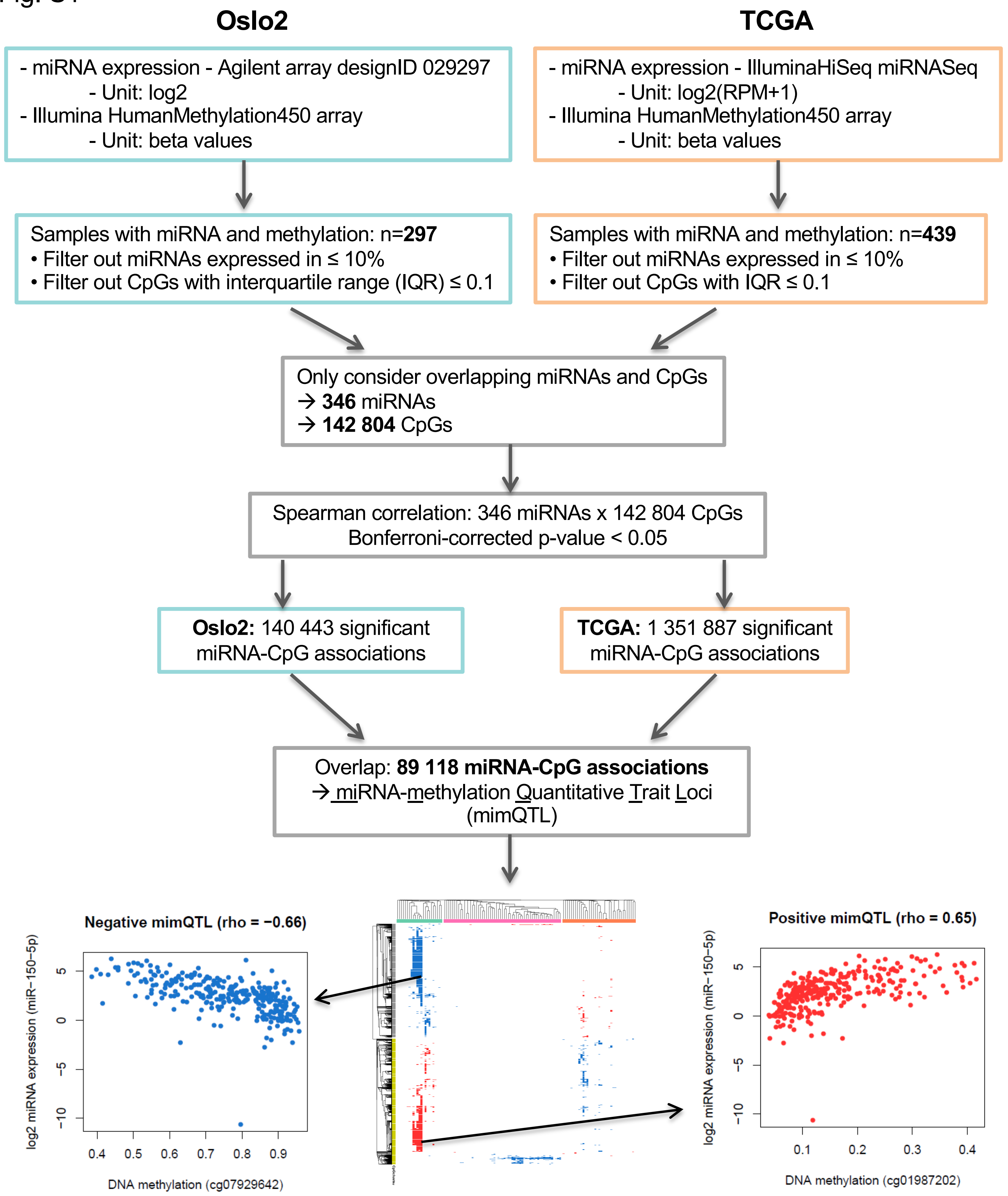

<u>Definitions</u>

***Cis* association**: Any association between a CpG and a miRNA located on the *same* chromosome

***Trans* association**: Any association between a CpG and a miRNA located on *different* chromosomes

Fig. S2

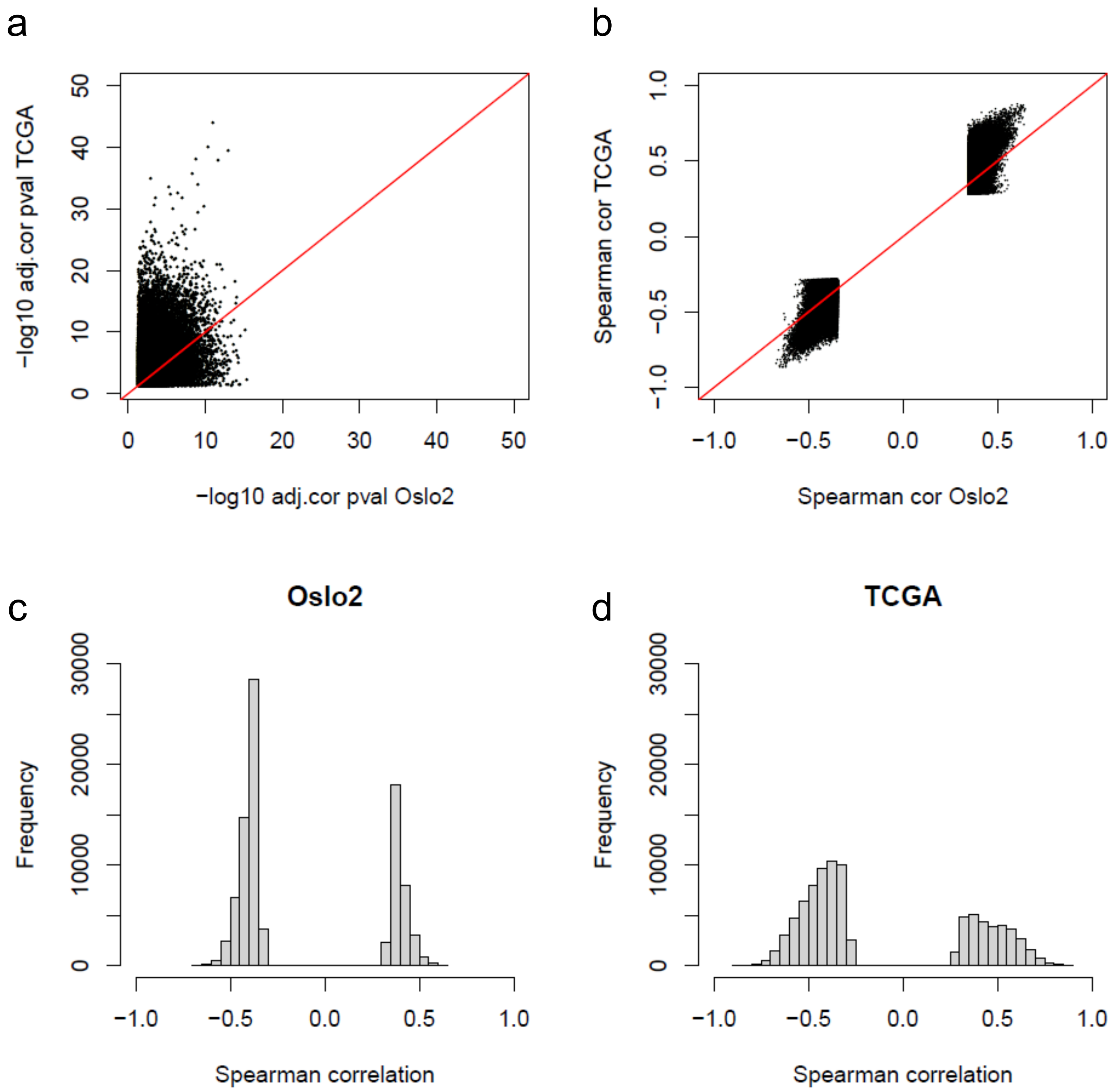

a
b
c
d
−log10 adj.cor pval TCGA
−log10 adj.cor pval Oslo2
0
10
20
30
40
50
Spearman cor TCGA
Spearman cor Oslo2
−1.0
−0.5
0.0
0.5
1.0
Oslo2
TCGA
Frequency
0
10000
20000
30000
Spearman correlation

Fig. S3

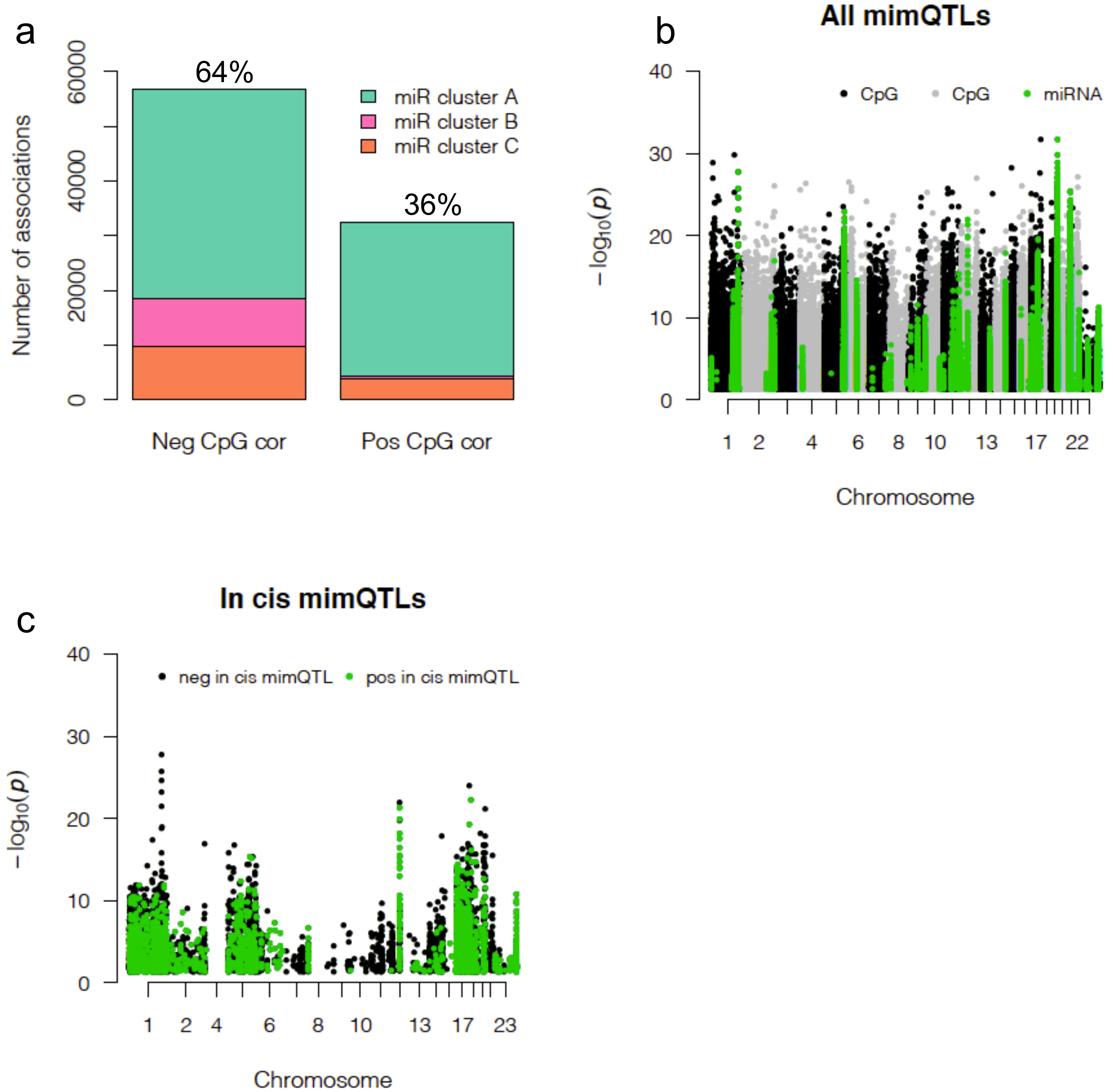
a
64%
36%
miR cluster A
miR cluster B
miR cluster C
Number of associations
0
20000
40000
60000
Neg CpG cor
Pos CpG cor
b
All mimQTLs
CpG
CpG
miRNA
– log10(p)
0
10
20
30
40
1 2 4 6 8 10 13 17 22
Chromosome
c
In cis mimQTLs
neg in cis mimQTL
pos in cis mimQTL
– log10(p)
0
10
20
30
40
1 2 4 6 8 10 13 17 23
Chromosome

Fig. S4

a

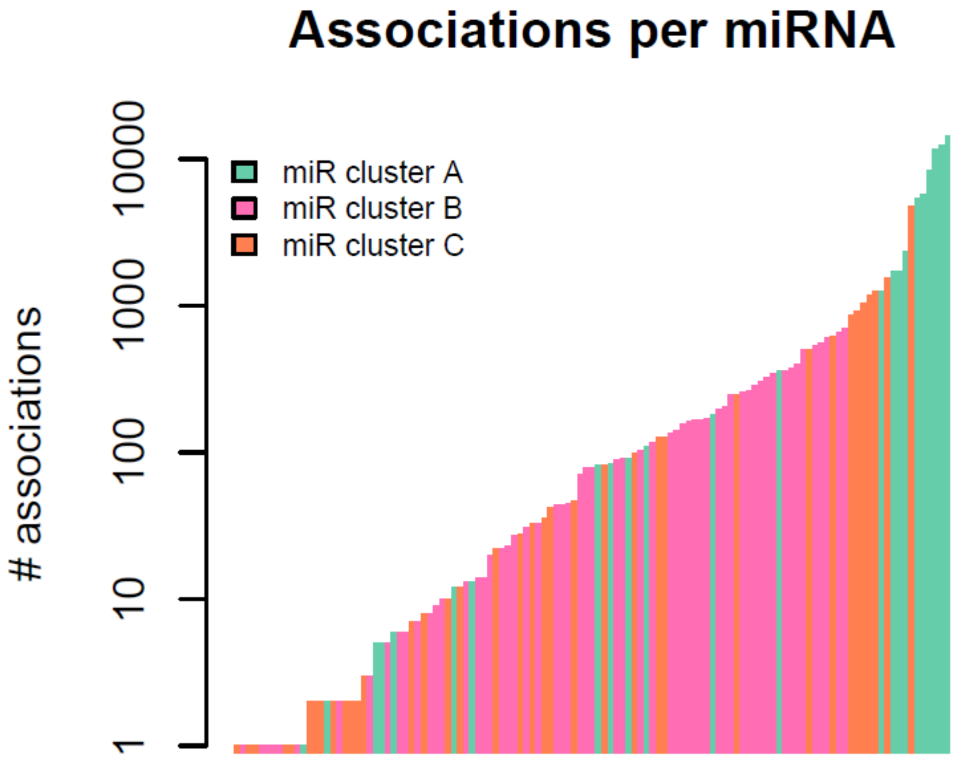

Associations per miRNA
associations
1
10
100
1000
10000
miR cluster A
miR cluster B
miR cluster C

b

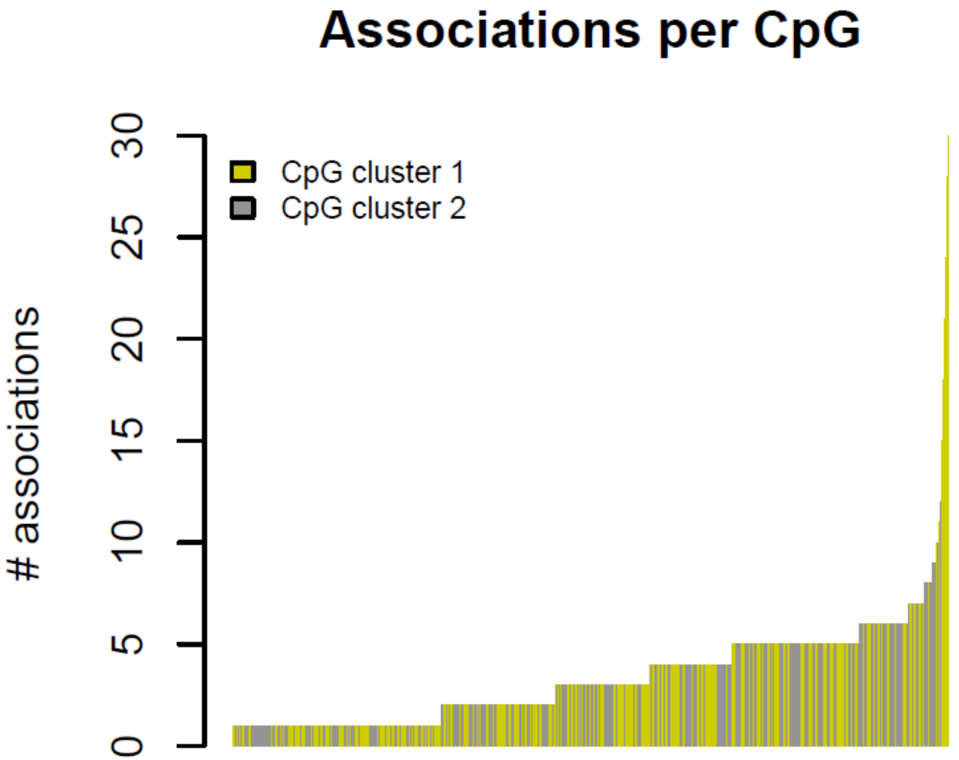

Associations per CpG
associations
0
5
10
15
20
25
30
CpG cluster 1
CpG cluster 2

Fig. S5

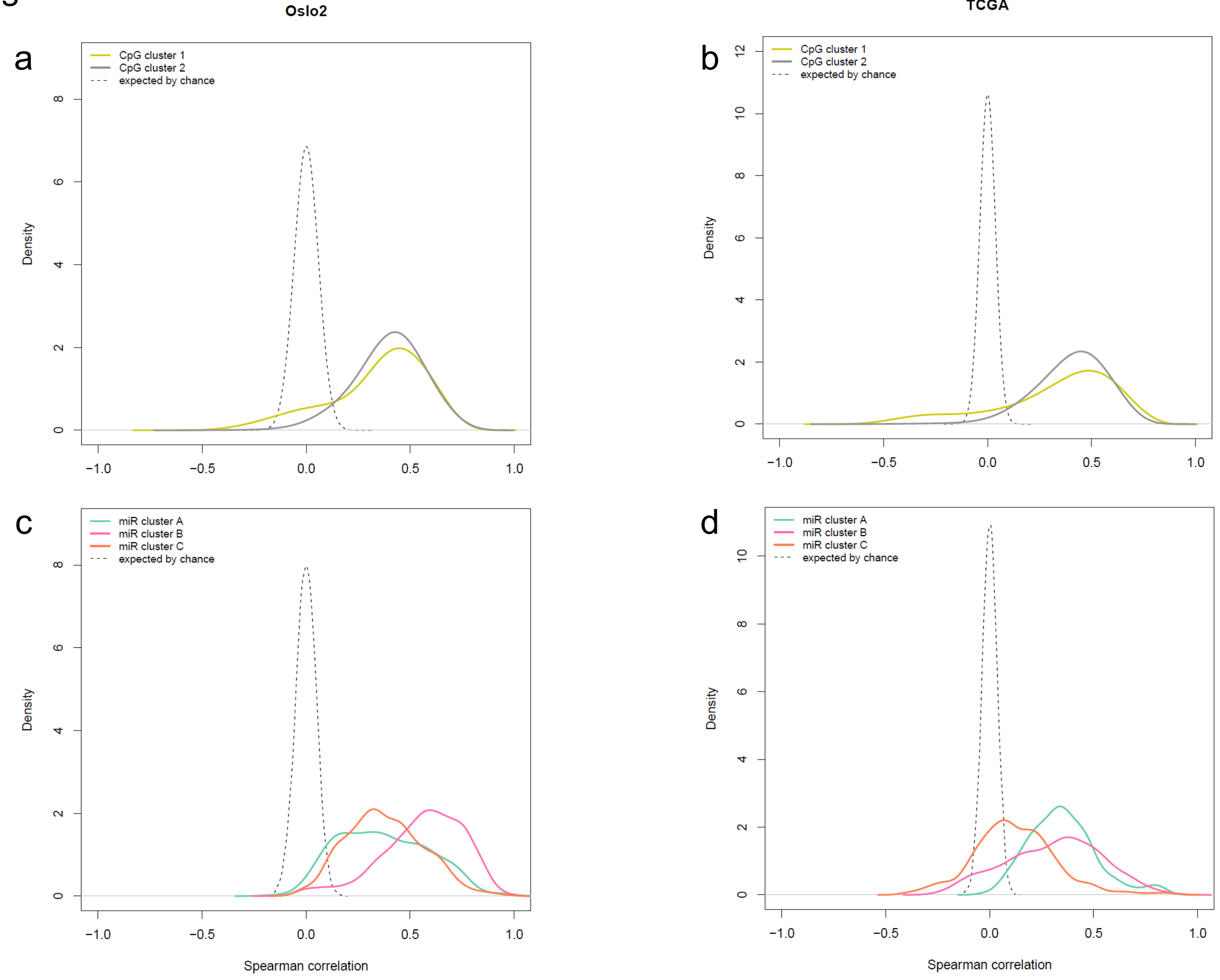

Oslo2
TCGA
a
b
c
d
CpG cluster 1
CpG cluster 2
expected by chance
CpG cluster 1
CpG cluster 2
expected by chance
miR cluster A
miR cluster B
miR cluster C
expected by chance
miR cluster A
miR cluster B
miR cluster C
expected by chance
Density
Density
Density
Density
Spearman correlation
Spearman correlation

Fig. S6

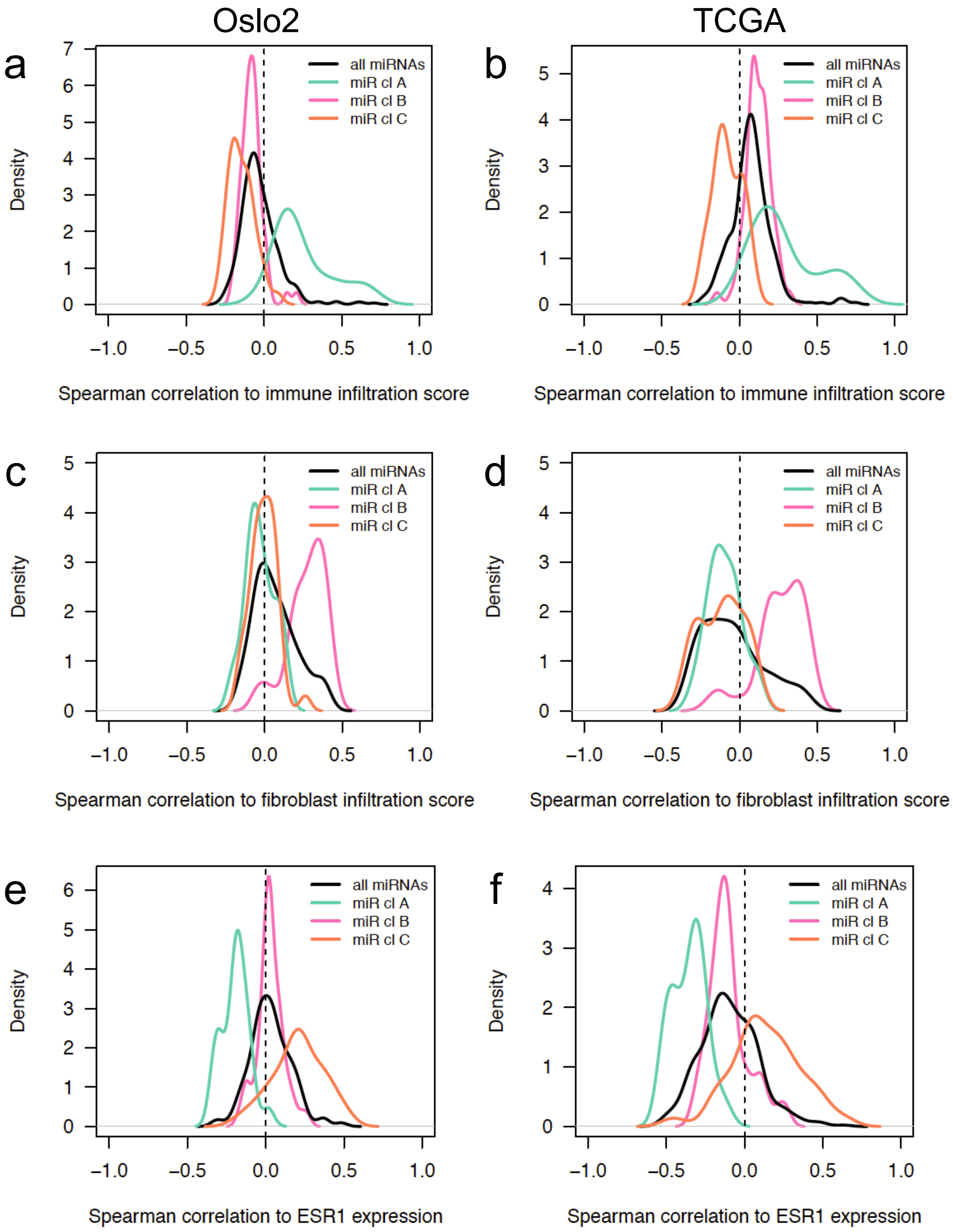

Oslo2
TCGA
a
b
c
d
e
f
Density
all miRNAs
miR cl A
miR cl B
miR cl C
−1.0
−0.5
0.0
0.5
1.0
Spearman correlation to immune infiltration score
Spearman correlation to fibroblast infiltration score
Spearman correlation to ESR1 expression

Fig. S7

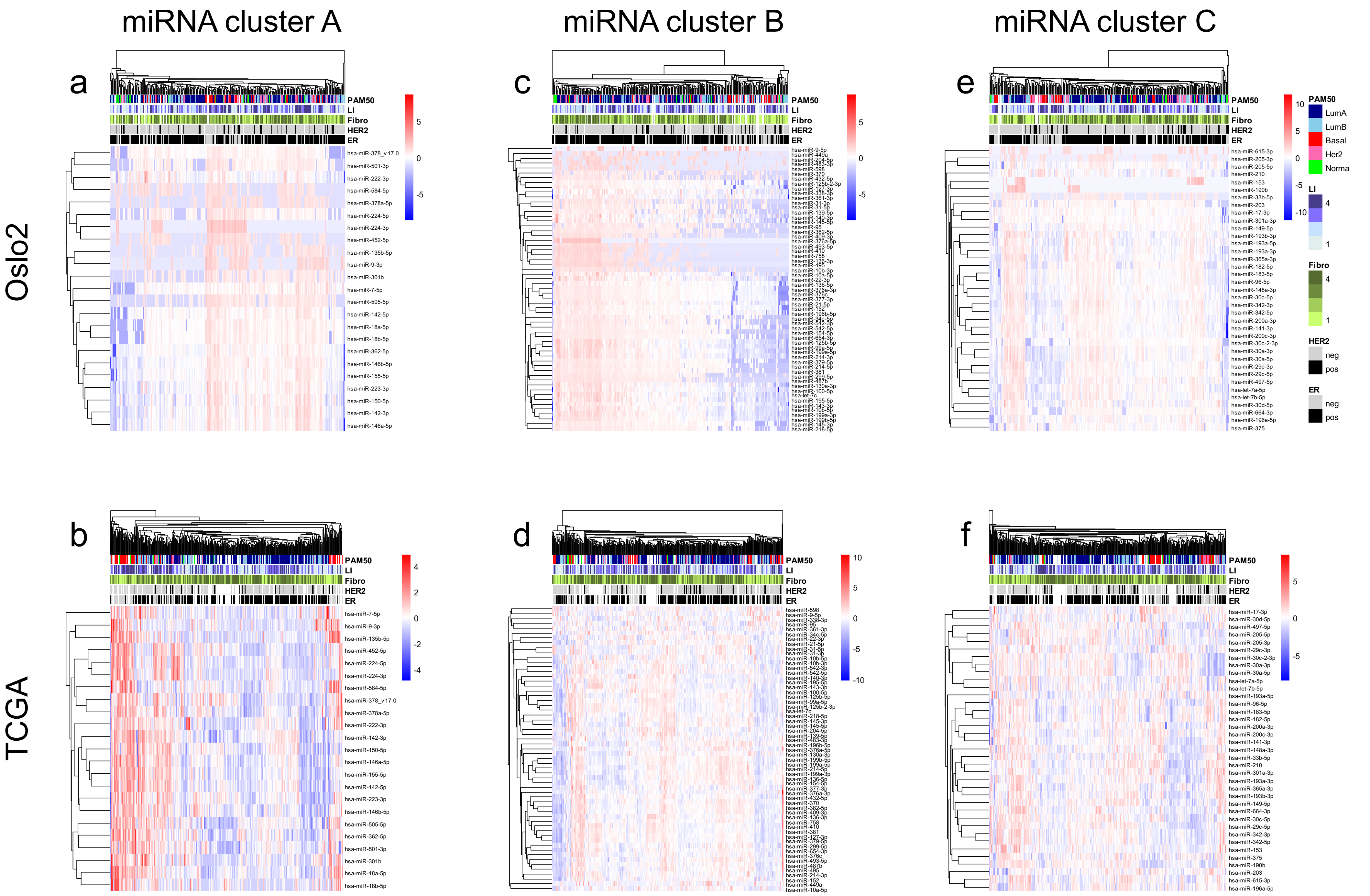
miRNA cluster A
miRNA cluster B
miRNA cluster C
a
b
c
d
e
f
Oslo2
TCGA
PAM50
LI
Fibro
HER2
ER
LumA
LumB
Basal
Her2
Norma
neg
pos

Fig. S8

a

CpG cluster 1
(TCGA)

b

CpG cluster 2
(TCGA)

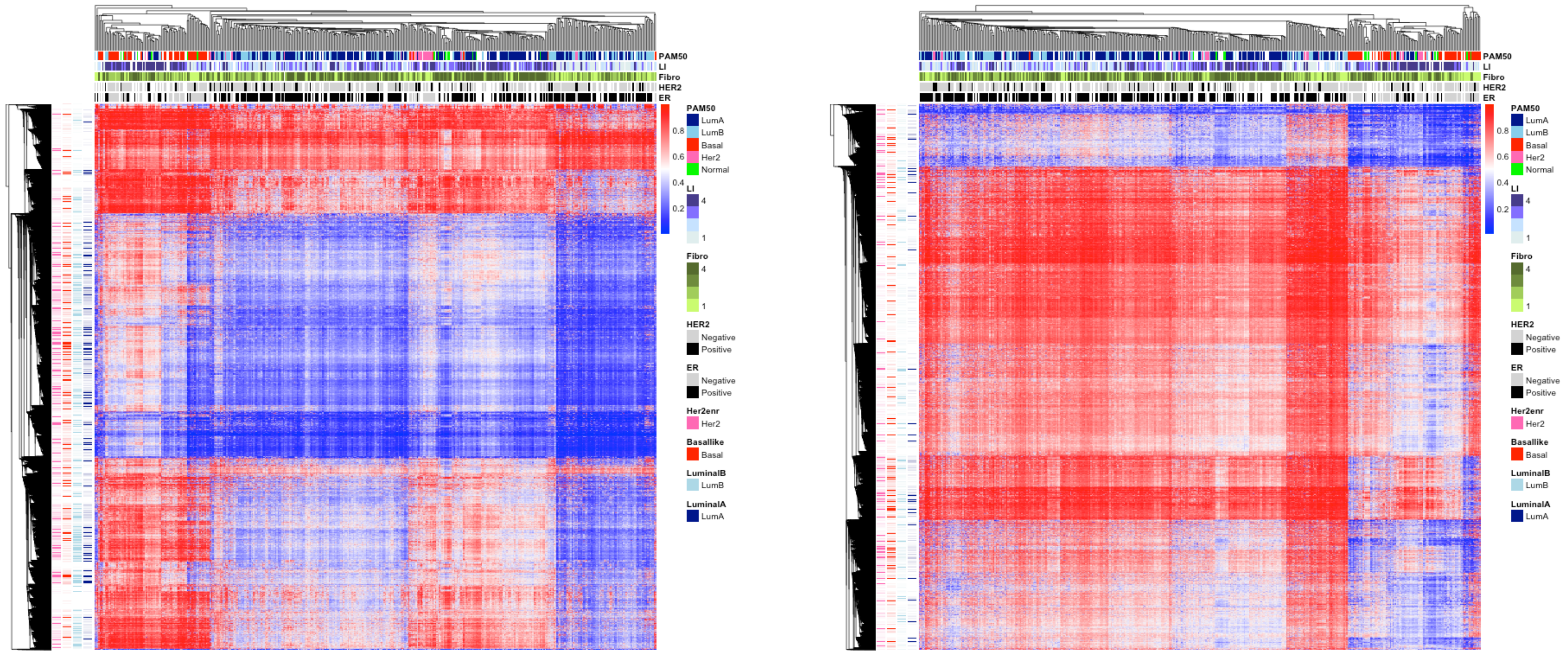

Fig. S9

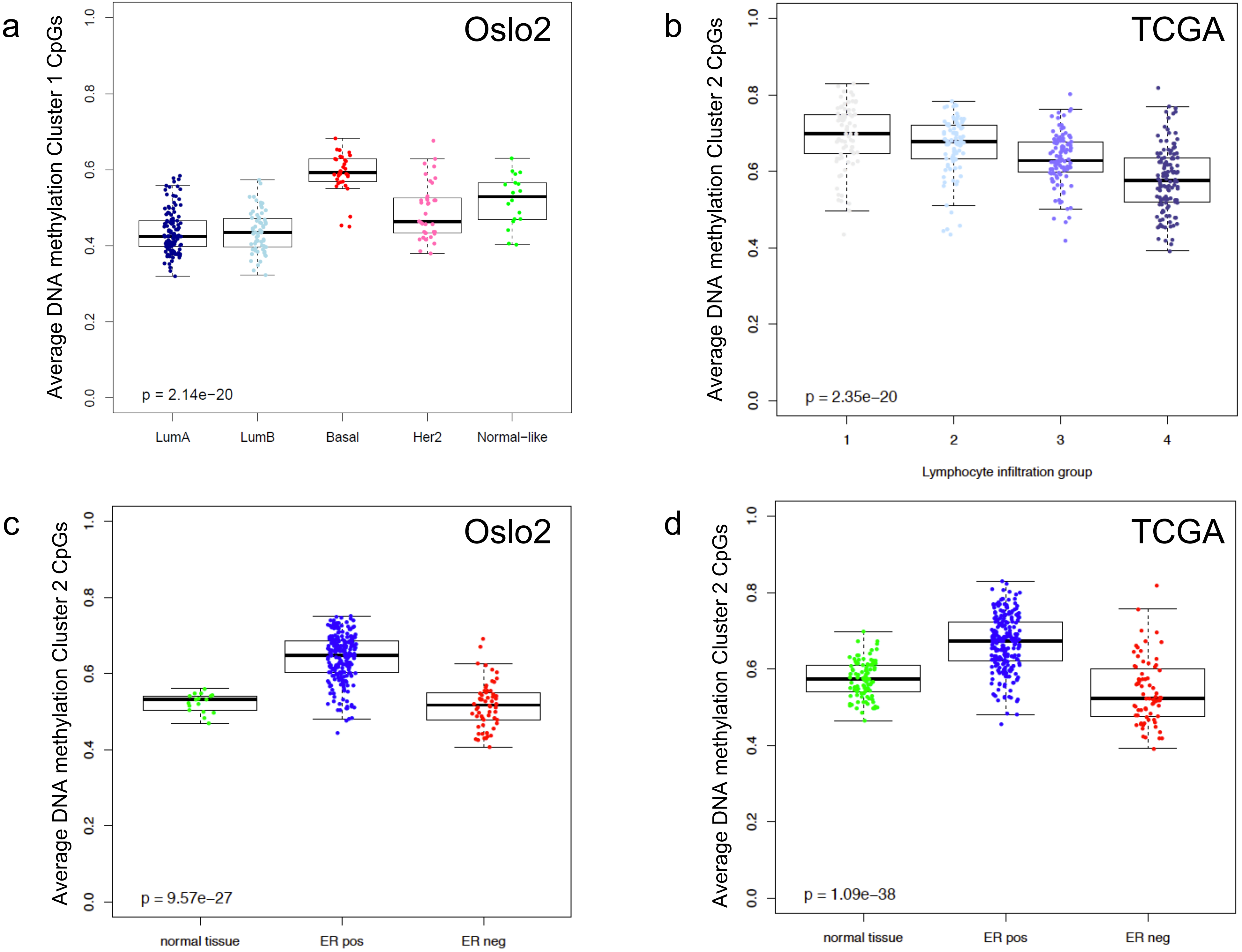
a
Oslo2
Average DNA methylation Cluster 1 CpGs
0.0
0.2
0.4
0.6
0.8
1.0
p = 2.14e−20
LumA
LumB
Basal
Her2
Normal−like
b
TCGA
Average DNA methylation Cluster 2 CpGs
0.0
0.2
0.4
0.6
0.8
1.0
p = 2.35e−20
1
2
3
4
Lymphocyte infiltration group
c
Oslo2
Average DNA methylation Cluster 2 CpGs
0.0
0.2
0.4
0.6
0.8
1.0
p = 9.57e−27
normal tissue
ER pos
ER neg
d
TCGA
Average DNA methylation Cluster 2 CpGs
0.0
0.2
0.4
0.6
0.8
1.0
p = 1.09e−38
normal tissue
ER pos
ER neg

Fig. S10

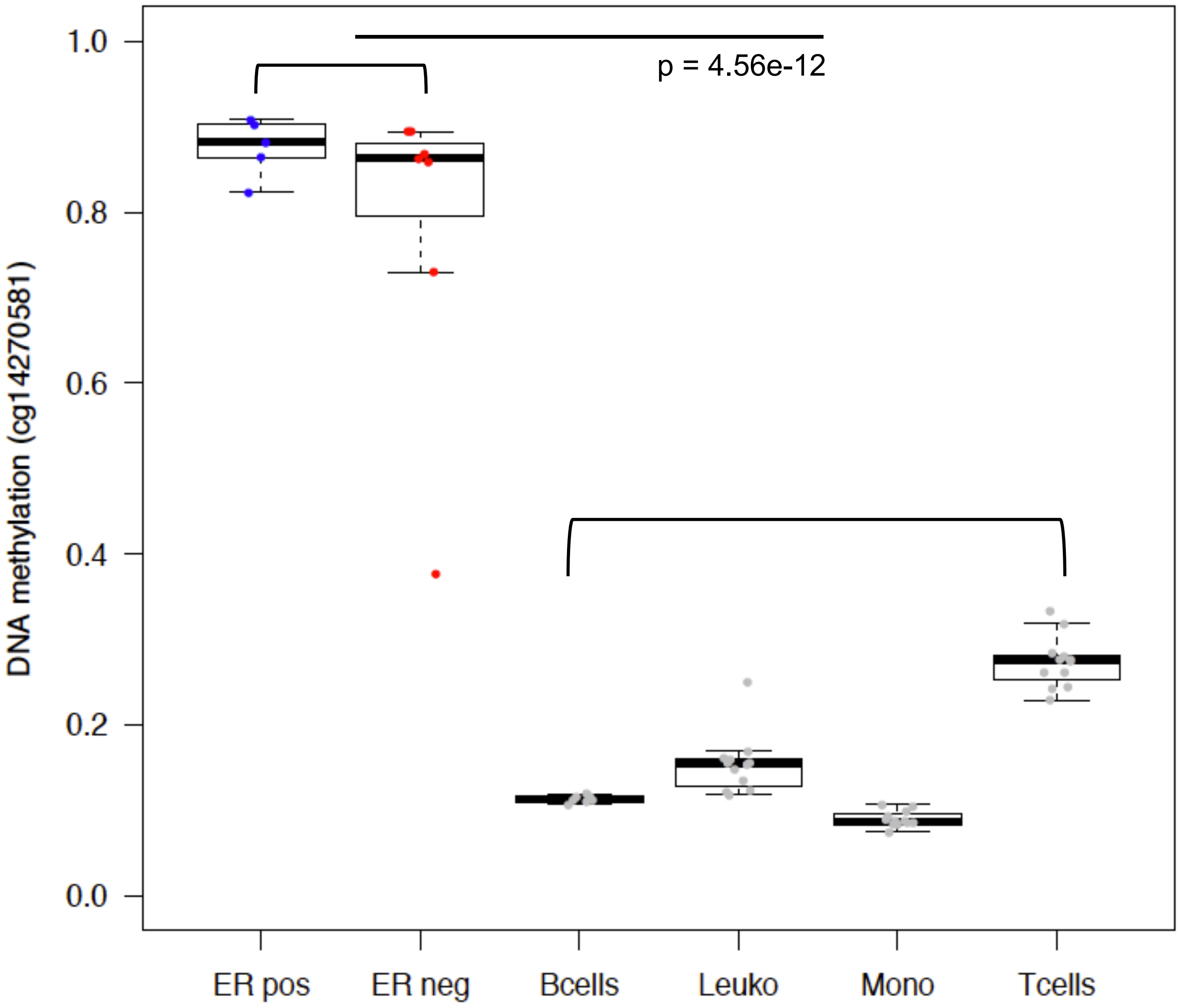

p = 4.56e-12
DNA methylation (cg14270581)
1.0
0.8
0.6
0.4
0.2
0.0
ER pos
ER neg
Bcells
Leuko
Mono
Tcells

Fig. S11

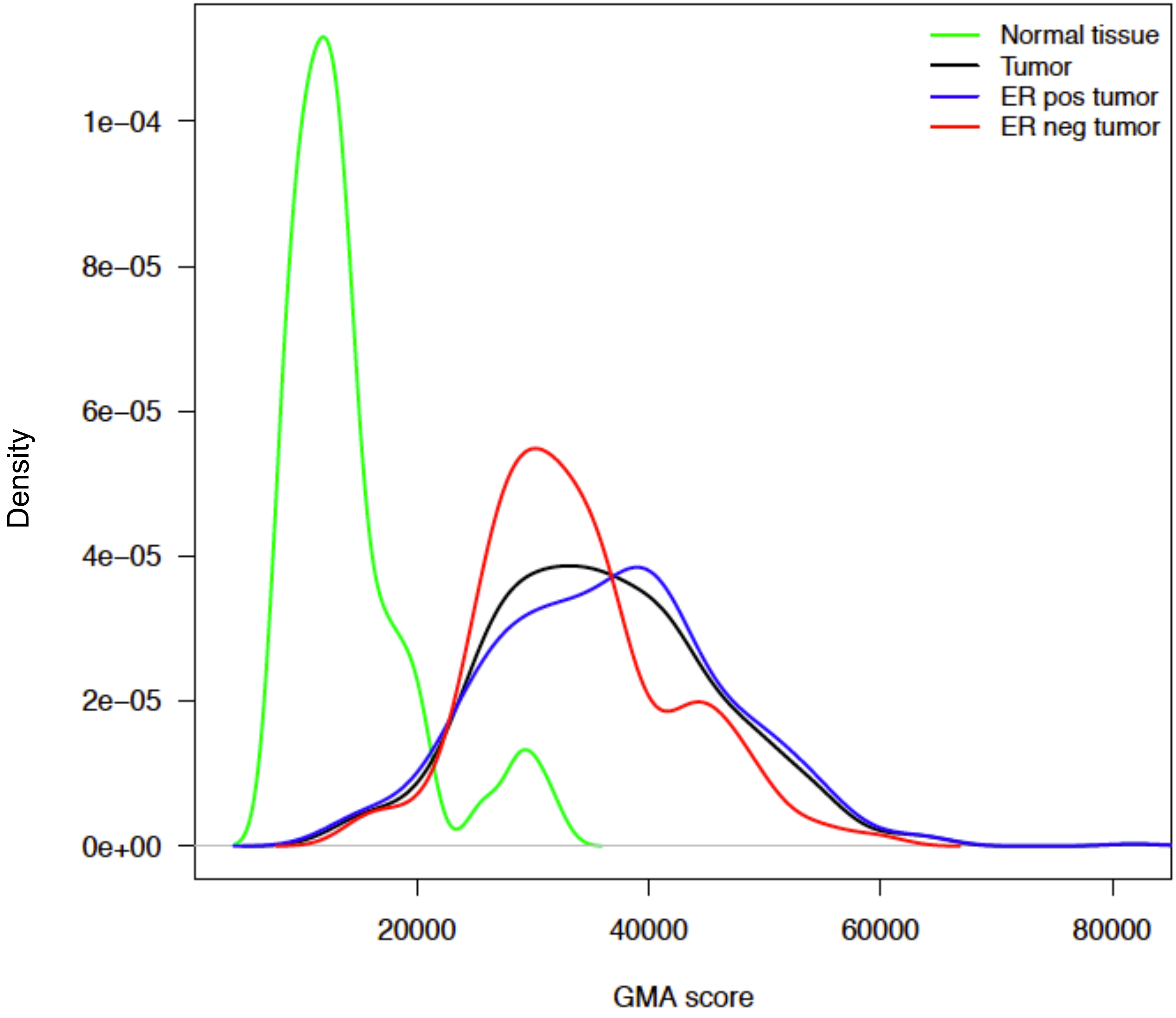

Normal tissue
Tumor
ER pos tumor
ER neg tumor
1e−04
8e−05
6e−05
4e−05
2e−05
0e+00
Density
20000
40000
60000
80000
GMA score

Fig. S12

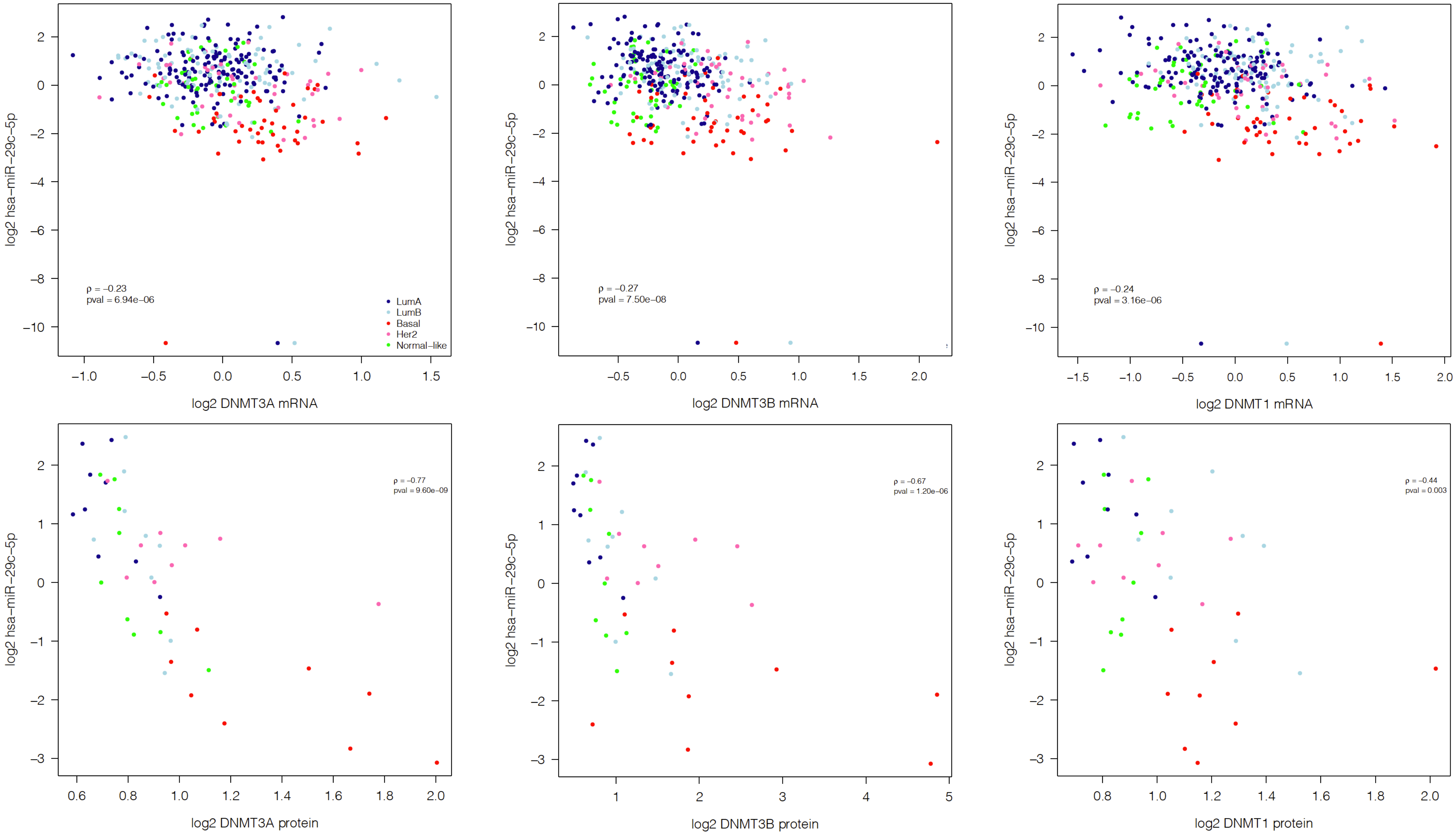
log2 hsa−miR−29c−5p
ρ = −0.23
pval = 6.94e−06
LumA
LumB
Basal
Her2
Normal−like
log2 DNMT3A mRNA
ρ = −0.27
pval = 7.50e−08
log2 DNMT3B mRNA
ρ = −0.24
pval = 3.16e−06
log2 DNMT1 mRNA
ρ = −0.77
pval = 9.60e−09
log2 DNMT3A protein
ρ = −0.67
pval = 1.20e−06
log2 DNMT3B protein
ρ = −0.44
pval = 0.003
log2 DNMT1 protein
